# Molecular tools, potential frontiers for enhancing salinity tolerance in rice: A critical review and future prospective

**DOI:** 10.3389/fpls.2022.966749

**Published:** 2022-07-28

**Authors:** Adnan Rasheed, Huijie Li, Muhammad Nawaz, Athar Mahmood, Muhammad Umair Hassan, Adnan Noor Shah, Fiaz Hussain, Saira Azmat, Syed Faheem Anjum Gillani, Yasir Majeed, Sameer H. Qari, Ziming Wu

**Affiliations:** ^1^Key Laboratory of Plant Physiology, Ecology and Genetic Breeding, Ministry of Education/College of Agronomy, Jiangxi Agricultural University, Nanchang, China; ^2^College of Humanity and Public Administration, Jiangxi Agricultural University, Nanchang, China; ^3^Department of Agricultural Engineering, Khwaja Fareed University of Engineering and Information Technology, Rahim Yar Khan, Pakistan; ^4^Department of Agronomy, University of Agriculture Faisalabad, Faisalabad, Pakistan; ^5^Research Center on Ecological Sciences, Jiangxi Agricultural University, Nanchang, China; ^6^Directorate of Agronomy, Ayub Agricultural Research Institute, Faisalabad, Pakistan; ^7^Department of Agriculture, Agriculture Extension and Adaptive Research, Government of the Punjab, Lahore, Pakistan; ^8^College of Agronomy, Gansu Agricultural University, Lanzhou, China; ^9^Department of Biology, Al-Jumum University College, Umm Al-Qura University, Makkah, Saudi Arabia

**Keywords:** rice, salinity stress, tolerance, CRISPR/Cas9, genes

## Abstract

Improvement of salinity tolerance in rice can minimize the stress-induced yield losses. Rice (*Oryza sativa*) is one of Asia’s most widely consumed crops, native to the subtropical regions, and is generally associated with sensitivity to salinity stress episodes. Salt-tolerant rice genotypes have been developed using conventional breeding methods; however, the success ratio is limited because of the complex nature of the trait and the high cost of development. The narrow genetic base of rice limited the success of conventional breeding methods. Hence, it is critical to launch the molecular tools for screening rice novel germplasm for salt-tolerant genes. In this regard, the latest molecular techniques like quantitative trait loci (QTL) mapping, genetic engineering (GE), transcription factors (TFs) analysis, and clustered regularly interspaced short palindromic repeats (CRISPR) are reliable for incorporating the salt tolerance in rice at the molecular level. Large-scale use of these potent genetic approaches leads to identifying and editing several genes/alleles, and QTL/genes are accountable for holding the genetic mechanism of salinity tolerance in rice. Continuous breeding practices resulted in a huge decline in rice genetic diversity, which is a great worry for global food security. However, molecular breeding tools are the only way to conserve genetic diversity by exploring wild germplasm for desired genes in salt tolerance breeding programs. In this review, we have compiled the logical evidences of successful applications of potent molecular tools for boosting salinity tolerance in rice, their limitations, and future prospects. This well-organized information would assist future researchers in understanding the genetic improvement of salinity tolerance in rice.

## Introduction

Rice is one of the most significant cereal crops and serves as a major dietary source and staple food for 50% of the world population, mostly in Asian countries (Rasheed et al., 2020a,b, 2021c,d). Due to its relatively small genome size, sufficient genetic diversity, molecular studies, and successful genetic transformation, rice has been labelled as a model crop (Chen et al., 2021b). The area under rice cultivation has been increased to counter the needs of the rapidly growing human population, which is expected to increase up to 9.5 billion by 2050 (Leridon, 2020). The goal of high-yielding rice must be attained (Kurniasih et al., 2021) under the increasing conditions of abiotic factors induced by climatic changes and uneven war for limited natural resources like water, land, and food (Park et al., 2022). The uneven distribution of natural resources pledged the war between rich and poor societies and enhanced hunger in many countries of the world, resulting in the loss of millions of human lives (Chen et al., 2021b). Abiotic stresses are a constant threat to agricultural sector and disturb the food supply chain (Rasheed et al., 2020c,d, 2021a,b). Salinity stress impairs rice growth, production, and yield and limits its areas of cultivation across many countries (Islam et al., 2021). Global estimates unleashed the fact that 1,000 million hectares of land is affected by salt stress, and besides this, around 30% of the irrigated land area is also disturbed (Shahid et al., 2018). This situation will be more worsen due to rise in seal level and rapidly growing human population (Liu et al., 2020a). Rice is considered a salt-sensitive crop, characterized by stunted plant growth, development, and yield loss when exposed to the salinity level of mmol L^−1^ NaCl (3 dSm^−1^) (Lutts et al., 1995). Recent research studies unravel that rice is more tolerant to salt stress during germination and vegetative stages than during seedling and reproductive phases (Bundó et al., 2022). Rice has adopted numerous strategies to cope with salinity stress at various developmental stages. Earlier researchers witnessed the 12% yield loss in rice at a salinity level of 3 dSm^−1^, and about a 50% yield drop has been recorded at a 6 dSm^−1^ salinity level (Linh et al., 2012).

In recent years, the development of salt-tolerant varieties and genetic studies to unlock the molecular mechanism of salinity tolerance have significantly contributed to the molecular breeding field (Niu et al., 2022). The development of high salt-tolerant varieties remains challenging because of several complications in plants’ response to salinity stress, which comprises ionic, and osmotic stress (Pareek et al., 2020). It has been a fact that salinity tolerance results from the coordinated action of several stress-responsive quantitative trait loci (QTL), genes, and enzymes induced by salt stress at various levels. The comprehensive mapping of potential QTL has been successful, but unfortunately, none of the identified QTL/genes is transformed into commercially cultivated rice cultivar (Kotula et al., 2020; Liu et al., 2020b). Salt stress has detrimental effects on rice growth, physiology, fertility, and yield, as shown by extensive research study (Tsai et al., 2019). Extensive research studies have underlined the underlying mechanisms of salt tolerance in rice, but they failed to provide a detailed overview of salt breeding in rice (Haque et al., 2021). For decades, rice breeders have been involved to cripple salt stress through multiple stress-related mechanisms (Chen et al., 2021b), but they did not shed light on the complete genetic control of salt tolerance (Prakash et al., 2022).

Therefore, breeding salt-tolerant rice varieties is the only promising step for ensuring food security in the future (Niu et al., 2022). Molecular studies have opened new doors to fully sequence rice genome and hunt for candidate genes in salt breeding programs (Habila et al., 2022). QTL and genes have shown tremendous results in developing tolerant varieties; however, further studies are needed to get a clear picture (de Ocampo et al., 2022). The introgression of QTL/genes encoding for salinity tolerance would compensate yield loss under salt stress (Prakash et al., 2022). Many TFs have been identified in rice contributing to salt tolerance and need to be transformed to develop transgenic rice varieties (Teng et al., 2022). A novel gene-editing tool, CRISPR/Cas9, emerged as the best-rewarded tool (Alam et al., 2022) that broke all biological barriers and showed the possibility of targeted gene editing for desired traits (Alam et al., 2022). Despite all of this, there is no sufficient progress in salt breeding in rice, and breeders are tackling this issue by adopting novel molecular tools leading toward sustainable agriculture (Fan et al., 2022). Secondly, the genetic diversity of rice is not fully conserved, which is constantly under threat because of the extreme climatic changes like rainfall, heat, fire, and land sliding (Mohammadinezhad et al., 2010). This review presented the latest developments about potent molecular tools used to sustain rice growth and yield under salt stress by identifying novel genes and their interacting networks. Molecular breeding tools have interfered in rice salt breeding programs and promised a more consistent result in coming time. We have debated the challenges and limitations of molecular studies and presented future prospective. This effort will be a powerful attempt to quicken the salt breeding program in rice.

## Effects of salinity stress on rice

**Figure 1:**
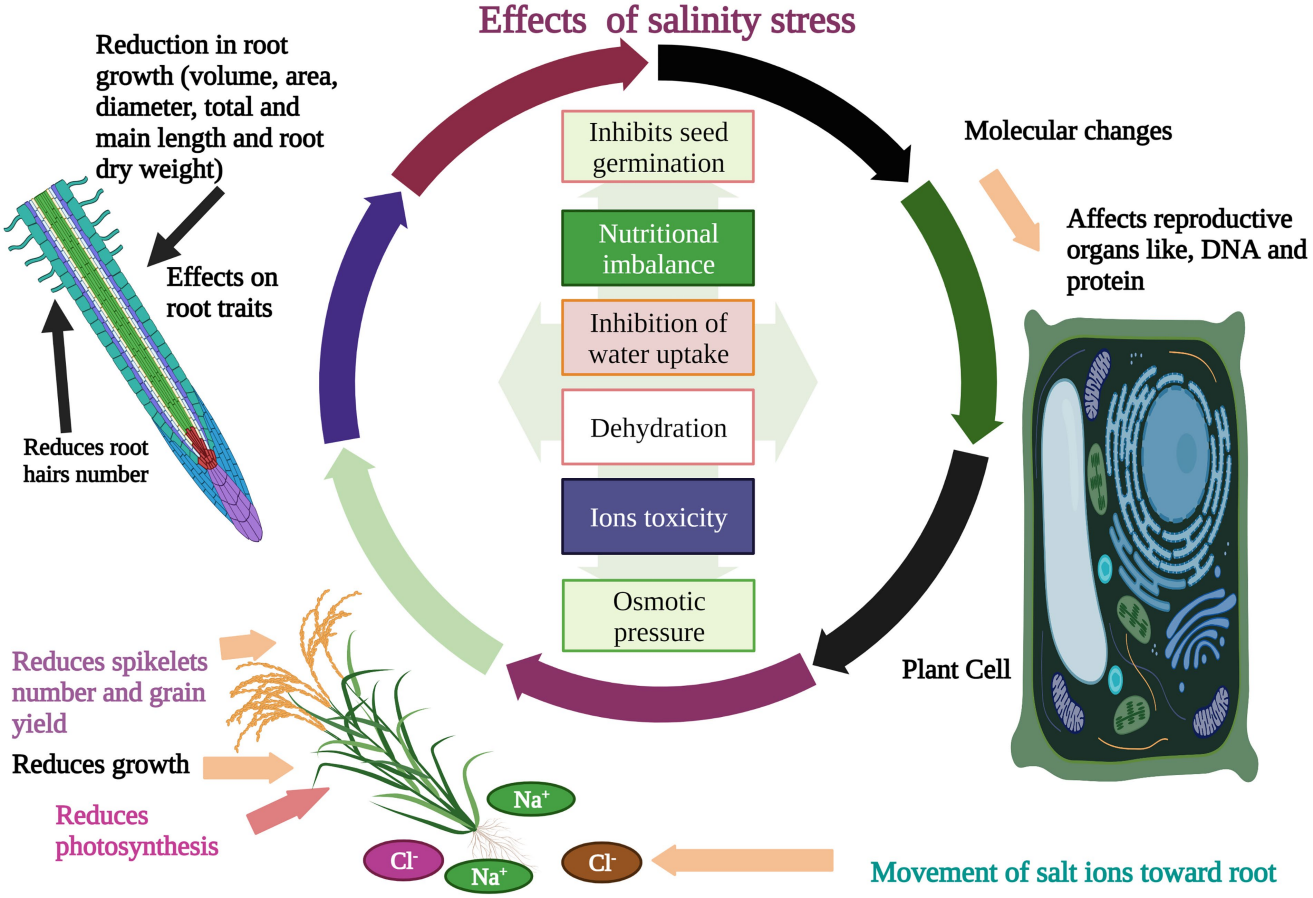
Effects of salinity stress on rice. Salinity stress affects rice seed germination, growth, and photosynthesis. Salinity stress induces ions toxicity, osmotic pressure, dehydration, and alteration in reproduction organs. Salinity stress also reduces roots hairs number, root volume, diameter, root area, length, root dry weight, spikelet’s fertility, grain yield, and nutrients uptake.

Salinity stress is one of the major impediments to rice production worldwide after drought (Zahra et al., 2022). Rice is relatively a highly salt-sensitive crop (Solis et al., 2022), as indicated by the rice threshold (dS/m) for salt stress (Maas, 1996). Rice seed germination is one of the main traits affected by salinity stress which causes poor seedling growth (Hasanuzzaman et al., 2009). Salinity stress impairs rice growth and production and leads to complete yield loss depending on its concentration and genotype. Salinity stress affected the rice plant height and tillers per plant when plants were exposed to 100 mM NaCl (sodium chloride; Soares et al., 2021). Salinity stress affected morpho-physiological traits and reduced dry matter yield (Channa et al., 2021). Phenolics and flavonoid contents are strongly affected by salinity stress in rice (Minh et al., 2016). Effects of salinity stress were observed on osmolytes and relative water contents (RWC) when a 7-days-old rice seedling was exposed to 100 mM NaCl. There has been a noticeable reduction in the contents of osmolytes (proline) under salinity stress (Polash et al., 2018). Salinity stress also affects rice sugar contents, pigments, and enzymatic activity (Amirjani, 2011). Hakim et al. (2014) evaluated five rice genotypes under salinity stress to observe the effects on growth (Figure 1), ion accumulation, and yield. Results showed that root length (RL), shoot length (SL), and yield traits were decreased with increasing salinity levels (Hakim et al., 2014). Higher salinity stress caused a huge decline in productive tillers and yield per plant in rice varieties (Arifuddin et al., 2021). Abdallah et al. (2016) exposed rice genotypes, Giza 177, and Giza 178 to different salinity levels (30, 60 mM NaCl). Salinity stress decreased photosynthesis pigments (Figure 1), carbohydrates, and activities of antioxidants like, SOD, APX, and CAT (Abdallah et al., 2016). Salinity stress reduced the transpiration rate, and stomatal conductance in rice. Salinity stress also caused the spikelet’s sterility, and reduced pollen viability (Irakoze et al., 2020). In another study, leaf number and total dry matter of rice were significantly reduced when rice genotypes were exposed to different salinity levels (50, 10, 150 mM) (Megha and Shankhdhar, 2022). Hence, effects of salinity stress on rice are harmful and caused significant yield loss which indicating a drift in global food security system. Effects of salinity stress could be minimized by evaluating rice genotypes against different levels of salt and observe the relative changes in studied characteristics.

## Mechanisms of salinity tolerance in rice

**Figure 2:**
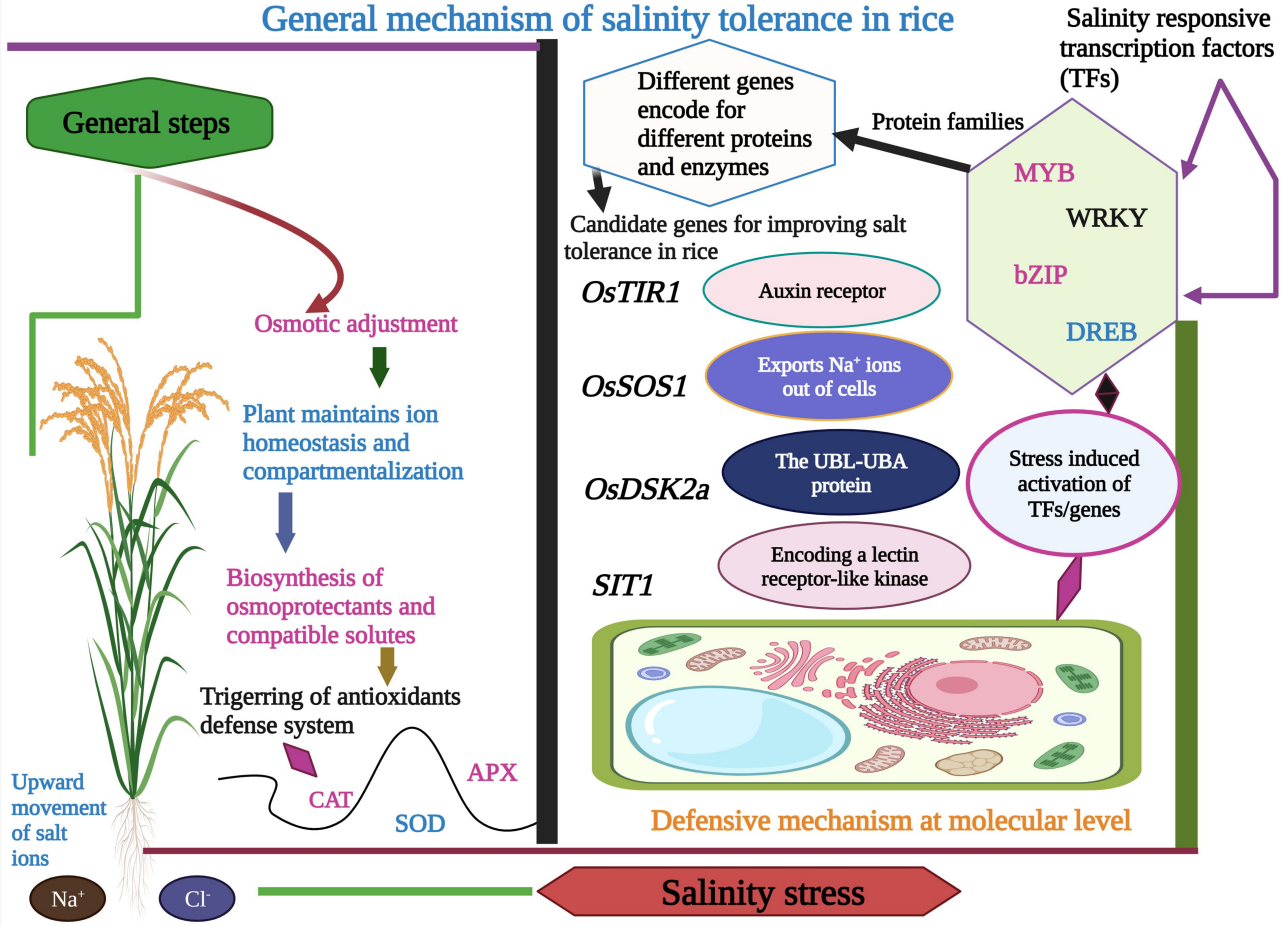
Graphical display of the different types of salt tolerance mechanisms in rice. It involves the activation of different salt-responsive TFs and genes which encode different proteins and enzymes. Besides this rice plant also activates the antioxidants defense system, maintains ions homeostasis, and synthesizes osmoprotectants and compatible solutes to counter the toxic effects of salinity stress.

To increase the rice yield under salinity stress, it is imperative to unfold the essential mechanisms involved in rice response to salinity stress (Reddy et al., 2017; Prakash et al., 2022). Studies recommended that multiple genes mainly control salinity tolerance in rice through a complex and interacting network (Chinnusamy et al., 2005; Wang et al., 2022; Yuan et al., 2022). Rice has been categorized in the list of salt-sensitive crops (Lutts et al., 1995; Sangwongchai et al., 2022), and salinity limits production at the maturity stage (Todaka et al., 2012; Kumar et al., 2022). Salinity stress could be alleviated at the seedling stage by removing old seedlings, but the rice cannot avoid stress during its flowering stage. The flowering stage is highly vulnerable to salinity stress and can cause an extraordinary loss in rice yield (Singh et al., 2004; Nagarajan et al., 2022), and salinity tolerance can be foreseen by comparing the percentage of biomass production under stress and non-stress conditions (Munns and Tester, 2008). Salt tolerance can be shown by two mechanisms, including ion exclusion and osmotic tolerance (Munns and Tester, 2008). These two salinity tolerance mechanisms are classified into ion exclusion, tissue tolerance, and osmotic tolerance or adjustment (Figure 2; Roy et al., 2014). The dominant mechanism is ions exclusion which involves the exclusion of toxic salt ions (Na^+^ and Cl^−^) from roots and prevents the uncontrollable concentration in the leaves. In this way, ions are retrieved from the xylem to the soil. The osmotic tolerance mechanism is controlled by a signaling network that compensate rice shoot growth, and it is triggered before ions accumulation (Rajendran et al., 2009).

Tissue tolerance involves the Na^+^ sequestration in the vacuole production of solutes and enzymes capable of detoxifying ROS. The genes involved in the tolerance mechanism are, *OsNHX, OsSOS1* which are functionally known as Na^+^/H^+^ antiporters (Kumari et al., 2013; Amin et al., 2016), *OsTPC1* (Ca^2+^ permeable network; Kurusu et al., 2012), *OsCLC1* (Cl^−^ network; Diédhiou and Golldack, 2006), and *OsNRT1;2* (nitrate carrier; Wang et al., 2012a). Besides this, early plant vigor is also used to characterize salinity tolerance in rice. Early growth mechanisms can avoid the toxic effect of salinity stress in rice (Kumari et al., 2013). To assess the balancing role of physiological traits in salinity tolerance, Yeo and Flowers (1990) evaluated the rice genotypes under salinity stress and measured the shoot Na^+^ concentration and plant vigor. Results showed that Na^+^ concentration in the shoot is lower in rapidly growing rice genotypes than in late-maturing genotypes. Growth vigor is an escaping strategy rather than a tolerance mechanism which is good as the yield is the main concern (Yeo and Flowers, 1990). Besides this different agronomic parameters play a key role in salinity tolerance in rice. In an experiment 12 rice cultivars were evaluated in a greenhouse against different levels of salinity stress. Noticeable genetic differences in relative salt tolerance were identified based on seedling growth. Genotypes were marked as salt-tolerant based on the average value of different traits. Dramatic changes in salt tolerance were noticed at early and seed maturing stages in genotypes (GZ5291-7-1-2 and GZ178). These results indicated that an early maturing attitude could prevent the toxic effects of salinity stress in rice (Zeng et al., 2002).

## Molecular factors contributing to salinity tolerance in rice

The genetic dissection of salt-tolerant regions is one of the most feasible approaches for developing salinity tolerance in rice (de Ocampo et al., 2022; Li et al., 2022b). One of such techniques is QTL mapping which allows the identification of genomic regions involved in salinity tolerance and ensures the effective introgression of candidate genes through marker-assisted selection (MAS; Singh et al., 2021; Dai et al., 2022). The development of salinity tolerant rice cultivars is suitable for growing on salt-affected soils across the globe (Ali et al., 2022; Prakash et al., 2022). For this reason, identifying hotspot regions associated with salinity tolerance is critical to be used in salt breeding programs. Hence, it has been proven that salt breeding programs cannot be successful without the interference of molecular tools (Fan et al., 2021; Yuan et al., 2022). Salinity stress affects the rice reproductive stage and undermines its yield and quality. However, few studies have been published on this aspect that strongly emphasized going into depth for a detailed investigation of changes and mechanisms (Singh et al., 2021).

## Quantitative trait loci mapping and genome-wide association studies for salinity tolerance in rice

Hundreds of potential QTL have been mapped for salinity tolerance in rice; however, few are cloned for transformation into elite cultivars. Recently, an F_2_ population was developed from cross of salt-tolerant (Kalarata) and salt-sensitive (Azucena) landraces. The F_3_ population was then evaluated and recorded for salt tolerance using several key indicators. Seedling growth, biomass, ions concentrations, and chlorophyll contents were studied. A total of 13 potential genomic regions were identified for 16 agronomic traits. Some novel QTL hold the key targets for QTL pyramiding to enhance salinity tolerance in rice (de Ocampo et al., 2022). In another study, a backcross inbred line population (BIL) was evaluated to unfold the genetic loci of a parent*. Oryza longistaminata* for salinity tolerance. As a result, 27 QTL were detected for salinity tolerance in 140 BIL. Results showed that 18 QTL were derived from *O. longistaminata*. The key indicators were salt injury score (SIC) and water content of seedling (WCS). QTL, *qSIS2*, and *qWCSST2* for these two traits were located on chromosome 2 (Yuan et al., 2022). Likewise, Le et al. (2021) studied QTL mapping for salt tolerance in Vietnamese rice landraces genotyped with 21,623 SNP markers. Plants were exposed to 100 mM of NaCl. Salinity tolerance traits like SIS, chlorophyll contents, water contents, and Na^+^ and Cl^−^ contents were studied. A total of 26 QTL were identified, and remarkably 10 of them were linked with various traits and involved in controlling several responses under salinity stress. Most of the QTL were co-localized with previously reported QTL (Le et al., 2021). The development of salinity tolerant rice varieties is limited because of the limited number of parental materials (Xie et al., 2021). A rice parent Haidao 86 (HD86), was evaluated to show the growth’s salinity tolerance, suggesting that it is a strong donor of salinity tolerant regions. An F_2_ segregating population was derived from a cross of HD86 with Nipponbare under 2% NaCl treatment. Salt-tolerant QTL, *qST1* was reported on chromosome 1 and explained 9.27% of genetic variance (Xie et al., 2021). Yadav et al. (2021) conducted a genome-wide analysis of the rice population to identify the marker-traits association. A total of 23 marker-traits associations were found for studied traits. They have two novel QTL, *qSDW2.1* and *qSNC5*, which could be a potential breeding target for salinity tolerance (Yadav et al., 2021). To unfold the genetics of salt tolerance at the reproductive stage, 140 F_2_ population was made by crossing PS5 a salt-sensitive and CSR10 a salt-tolerant genotype. Salinity tolerance was assessed using several morphological, and biochemical traits. A total of 39 QTL with explained variance of 3–45% were identified. Major and significant QTL were identified for sodium content, potassium content, and sodium/potassium ratio in leaves and shoots. The QTL, *qNaL-1.2*, *qKR-1*, and *qNa/KL-1.2* with 45, 35, and 32% of phenotypic variance were mapped on chromosome 1. Beside this a novel QTL, *qGY-2* for grain yield was identified on chromosome 2. QTL validation may be advantageous for MAS breeding for development of commercially important salt-tolerant cultivars (Pundir et al., 2021).

Salinity tolerance at the rice bud stage directly affects the seedling and yield; however, QTL mapping for salinity tolerance at the bud burst stage is limited. Earlier, a recombinant inbred lines (RIL) population was developed by crossing salt-sensitive (Kongyu131) and salt-tolerant cultivars (Xiaobaijingzi) and used to map the major QTL for salinity tolerance at the bud burst stage using the high-density genetic map. Two QTL, *qRSL3*, and *qRRL3* were mapped on chromosome 3 and related to salinity tolerance (Lei et al., 2021). Rice is highly sensitive to salinity stress at the seedling and germination stage. QTL mapping for salinity tolerance at the seedling stage can prevent yield loss. Nakhla et al. (2021) evaluated the BIL population and conducted QTL mapping analysis for several traits at the seedling stage. They have identified 46 loci at the seedling stage. The identified 9 loci and 33 loci with ACC9 parent alleles increased salinity tolerance at the seedling stage. This locus can improve salinity tolerance in rice breeding programs (Nakhla et al., 2021). Kumari et al. (2021) identified several QTL for salinity tolerance in rice using the back cross population (BC_1_F_2_). QTL, *qSTY11.1* for seedling salinity tolerance showed a promising increase in salinity tolerance in the BC population (Kumari et al., 2021).

In the same way, to study the genetic variation among the rice landraces, QTL mapping was conducted to identify the rice tolerance to salinity stress. 13 QTL were mapped out of them, ten were aligned with previously reported QTL (Alam et al., 2021). Hence an appropriate breeding plan is critical to developing salinity tolerance in rice by QTL pyramiding. A genome-wide association study (GWAS) was applied to identify rice QTL linked with salinity tolerance. A diverse rice panel was exposed to 100 mM NaCl to investigate the significant difference in salinity tolerance and identify the potent genomic regions. QTL, *qSLn1.1,* and *qRLs2.1* were identified for shoot length and root length positioned on chromosomes 1 and 2 (Nayyeripasand et al., 2021). GWAS is one of the most powerful techniques which provide the marker traits association and identify the potential QTL for salinity tolerance. Therefore, it is critical to conduct GWAS better to understand the genetic control of salinity tolerance in rice. A multiparent advanced generation intercross (MAGIC) population was used to identify the novel QTL underlying salinity tolerance in rice. The candidate gene analysis discovered 12 genes, including SCKI, for rice salinity tolerance under NaCl conditions (Krishnamurthy et al., 2022). Several studies have uncovered the genomic regions for salinity tolerance in rice; however, a complete genetic understanding of salinity tolerance is still a mystery. Among all population, RILs is the most feasible for QTL mapping. Another study conducted by Kong et al. (2021) identified the QTL for survival traits in the rice RILs population. They have identified seven QTL positioned on chromosomes 3, 4, 5, 6, and 8 for several traits. Among the identified QTL, *qST-3.1*, *qST-5.1*, *qST-6.1*, and *qST-6.2* (Table 1) were new in this study, and their contribution to salinity tolerance was functionally confirmed by relative analysis of RILs. The gene combination result of these four new QTL highlighted that the mixture of the four QTL cooperative genotypes could meaningfully enhance the salinity tolerance in rice (Kong et al., 2021). Yield is an end product of any breeding program. Salinity stress greatly affects the yield of rice in salt-affected areas. In experiment, 42 chromosomal segment substitution lines (CSSLs) were evaluated under salinity stress to map the potential genomic regions for yield and yield components. A total of six QTL were identified for grain yield and yield-related traits located on chromosome 2. A novel QTL, *qSTGF2* for grain filling trait, was considered a potent donor to higher grain yield under extreme salinity stress in CSSLs (Mai et al., 2021). Another major QTL, *qRSL7*, for the bud burst stage, was identified in the F_2:3_ population. This major QTL was detected on chromosome 7, underlying shoot length-based salinity tolerance in rice (Lei et al., 2020). Another RIL population was used to identify the QTL underlying salinity tolerance in rice and the relationship between salt-induced injury and changes in different physiological and biochemical traits. In total 23 QTL were identified, including the earlier reported QTL *Saltol*. The QTL for salt injury score (SIS) were detected on chromosomes 1, 4, and 12 and linked with Na^+^ transport from roots to shoots in the rice population. The QTL, *qSIS1* showed an additive effect and can be effectively used in QTL pyramiding (Chen et al., 2020).

**Table 1:** List of several salt-tolerant QTL in rice identified by GWAS and QTL mapping.

Population/parents	Trait	QTL	Chromosome	References
Backcross inbred lines (BIL)	Salt injury score and water content of seedling	*qSIS2*, *qWCSST2*	2	Yuan et al., 2022
F_2_ population (Haidao 86, Nipponbare)	Salt tolerance	*qST1*	1	Xie et al., 2021
96 landraces along with FL478 as tolerant and IR29 as susceptible check	Shoot dry weight, and shoot Na^+^ contents	*qSDW2.1* and *qSNC5*	2, 5	Yadav et al., 2021
140 F_2_ population (PS5, salt sensitive, CSR10, salt tolerant variety)	Sodium contents, potassium contents, sodium/potassium ration, and grain yield.	*qNaL-1.2*, *qKR-1*, and *qNa/KL-1.2, qGY-2*	1, 2	Pundir et al., 2021
195 RIL (Salt sensitive, Kongyu131, salt-tolerant cultivar., Xiaobaijingzi)	Relative shoot length and relative root length.	*qRSL3*, *qRRL3*	3	Lei et al., 2021
BIL population (ACC9 as donor and Zhenshan97 as a recurrent parent)	Seedling height and sodium contents	*qSH1, qNa2.1*	1, 2	Nakhla et al., 2021
BC_1_F_2_ (NKSWR 173, IR 64)	Seedling salinity tolerance	*qSTY11.1*	11	Kumari et al., 2021
176 rice landraces	Plant shoot length/biomass	*qCDP1.1*	1	Alam et al., 2021
Diverse rice panel	Shoot length, root length, root fresh weight	*qSLn1.1, qRLs2.1, qRFWs6.1*	1, 2, 6	Nayyeripasand et al., 2021
160 RILs (Luohui 9, X RPY geng)	Salt tolerance traits	*qST-3.1*, *qST-5.1*, *qST-6.1*, and *qST-6.2*	3, 5, 6	Kong et al., 2021
42 IR64-CSSLs	Grain yield, grains filling	*qSTGY2.2, qSTGF2*	2	Mai et al., 2021
F2:3 (IR36 salt-sensitive, Weiguo salt-tolerant)	Relative shoot length	*qRSL7*	7	Lei et al., 2020
RIL (LYP9 and PA64s)	Shoot length	*qSL7*	7	Jahan et al., 2020
148 RIL (IR29 salt-sensitive Pokkali salt-tolerant)	Salt injury score	*qSIS1*	1	Chen et al., 2020
100 RIL	Salinity survival index and shoot length	*qSSI1, qSL1*	1	Dahanayaka et al., 2017
ILs	Salinity injury score	*qSIS1.39*	1	De Leon et al., 2017
BC_1_F_5_ ILs (introgression lines)	Chlorophyll content	*qChlo4*	4	Pang et al., 2017
300 F_5:6_ RIL	Root length	*qRL6.1, qRL12.1*	6, 12	Bizimana et al., 2017
600 RIL	Root length	*qRL1.2*	1	Rahman et al., 2017
F_2_ (Pollaki, salt-tolerant and IR36, salt-sensitive).	Grain length-width ratio	*qGLWR2*	2	Khan et al., 2016
216 RIL	Grain yield	*qSSIGY2.1*	2	Tiwari et al., 2016
Introgression lines (ILs)	Spikelet’s fertility	*qSF1.4*	1	Lu et al., 2014
RIL (Jiucaiqing, IR26)	Shoot height and dry shoot weight	*SH12.1*, *qSH12.2*, and *qDSW12.1*	12	Wang et al., 2012b

Jahan et al. (2020) used a RIL population developed from a cross of LYP9 and PA64s and showed significant variation for morphological traits under 100 mM salinity stress. 38 QTL for six variables were detected on chromosomes 1, 2, 3, 4, 5, 6, 7, and 10 under different salinity conditions. A novel QTL, *qSL7* (Table 1), was identified on chromosome 7 under two different salinity conditions (Jahan et al., 2020). In another study, three major QTL, *SH12.1*, *qSH12.2*, and *qDSW12.1* were identified for seedling height (SH) and dry shoot weight (DSW) in the RIL population exposed to 0.7% NaCl for 10 days (Wang et al., 2012b). One of the key methods for rapid identification of QTL is bulk segregant analysis. Tiwari et al. (2016) used RIL population grown under salinity stress and reported 34 QTL; out of them, several QTL were novel which ensured the success of QTL pyramiding (Tiwari et al., 2016). Many QTL have been identified using elite breeding population like RIL. RIL population was exposed to 100 mM of NaCl which induced noticeable changes in morpho-physiological traits. Six QTL were identified on chromosome 1, and 4 out of them *qSSI1*, *qSL1* were identified for salinity survival index (SSI) and shoot length (SL; Dahanayaka et al., 2017). Identification of stress-related QTL increased our understanding of the complex nature of salinity tolerance. Different studies showed different results; however, the nature and pattern of salt stress are the same. Khan et al. (2016) investigated salt tolerance in rice using F_2_ population made by crossing Pollaki (salt tolerant), and IR36 (salt sensitive). The population was exposed to different salt stress conditions. They have identified six QTL for different agronomic traits (Khan et al., 2016). Pang et al. (2017) conducted QTL pyramiding to develop super high rice with improved tolerance to salinity stress. Out of many identified QTL, *qChlo4* (Table 1) was detected for chlorophyll content which demonstrated that QTL pyramiding is a powerful way of genetic dissection of a complex trait of salinity tolerance in rice (Pang et al., 2017). In another study, 300 F_5:6_ RIL population was used to identify the QTL for salinity tolerance in rice. Twenty novel QTL were mapped on chromosomes 1, 3, 4, 6, 8, 9, and 12. It is strongly suggested to integrate the QTL into one genetic background by QTL pyramiding to enhance the salinity tolerance in rice (Bizimana et al., 2017). In a recent study, Krishnamurthy et al. (2020b) successfully introgressed the Satlol QTL in two high-yielding rice cultivars (Pusa44 and Sarjoo52) using the marker-assisted backcrossing breeding (MABB) technique. These techniques showed an improvement in salinity tolerance and the development of tolerant lines with the genetic background of Pusa44 and Sarjoo52 (Krishnamurthy et al., 2020b). The detailed and comprehensive analysis of salinity tolerance in rice suggested that salinity tolerance is a complex trait and needs further studies and use of emerging molecular techniques to develop tolerant rice cultivars. Biochemical based salinity tolerance in rice is poorly understood and needs further investigation. QTL and GWAS will unfold several new mechanisms of salinity tolerance in rice which is a critical need of time.

## Application of CRISPR/Cas9 for salinity tolerance in rice

**Figure 3:**
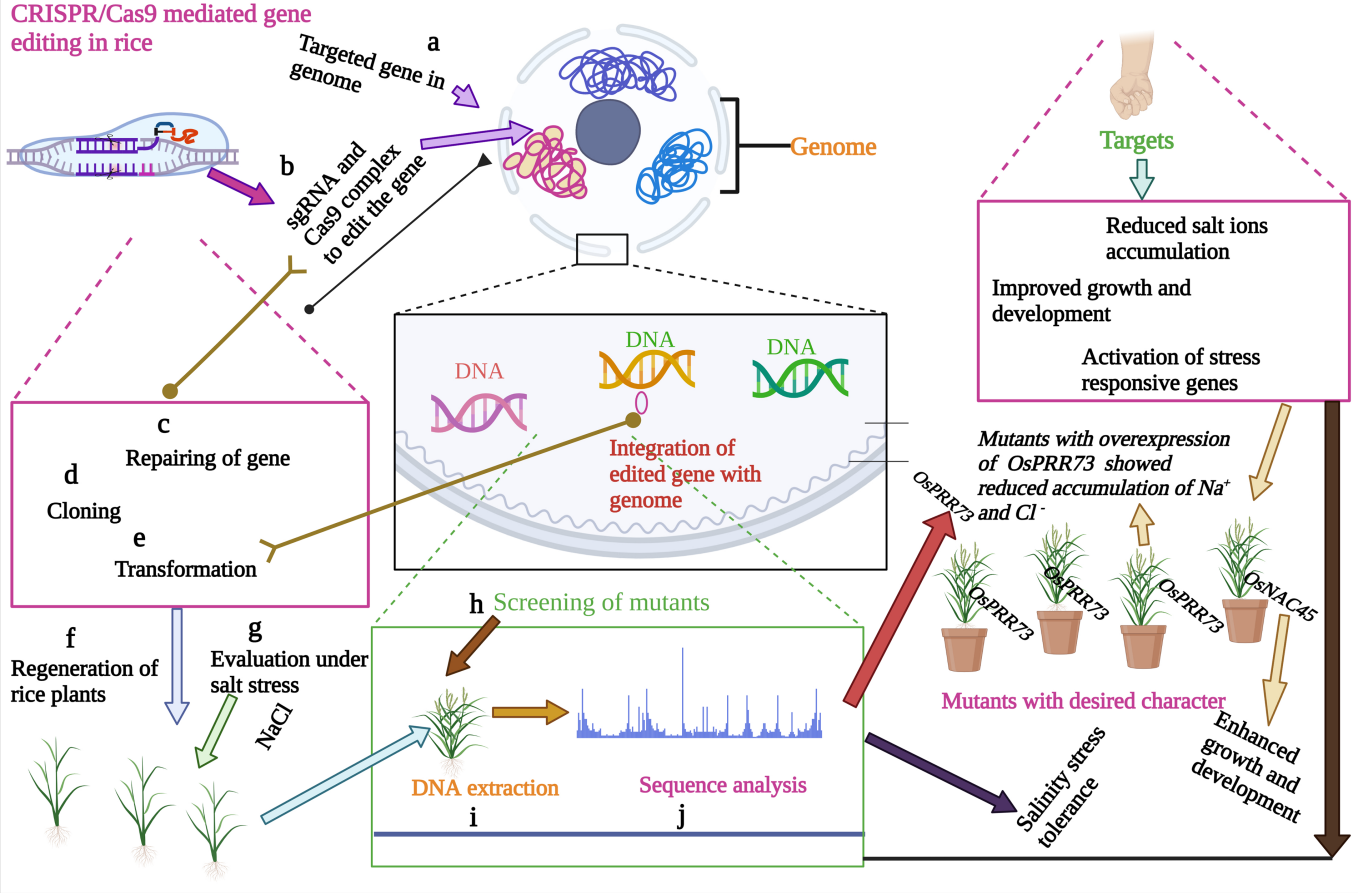
Key steps of CRISPR/Cas9 mediated gene editing for salinity tolerance in rice. CRISPR/Cas9 has the ability to edit the gene of interest for salinity tolerance by a complex mechanism. Mutants plants with salinity tolerance character are exposed to salinity stress to confirm the targeted gene expression.

CRISPR/Cas9 gene-editing technique has been effectively used for targeted gene editing to enhance salinity tolerance in rice (Zhang et al., 2019a; Zegeye et al., 2022; Zhou et al., 2022; Figure 3). Targeted editing of salt-tolerant genes has been achieved (Nazir et al., 2022). Since the CRISPR/Cas9 tool emerged, speed of salinity tolerant rice cultivar development has been enhanced. Nevertheless, the underlying molecular mechanisms and individual genes involved in rice salinity tolerance are illuminated. Han et al. (2022) employed the CRISPR/Cas9 to edit the *OsRR22* gene, which controls the salinity tolerance in rice. The mutant plants showed considerable differences in plant height and total fresh weight under two different salt concentrations (Han et al., 2022). Another salt-tolerant gene, *OsDST*, was edited by CRISPR/Cas9, which showed significant improvement in salinity tolerance in the indica elite cultivar. Mutants showed a high level of salinity tolerance at the seedling stage (Santosh Kumar et al., 2020). In another study, Zhang et al. (2019b) demonstrated targeted editing of *OsOTS1* in rice by CRISPR/Cas9. CRISPR/Cas9 induced efficient gene knockout with 95% of transgenic plants exhibited the desired results with zero off-target effects (Zhang et al., 2019b). Different TFs have been edited to enhance salinity tolerance in rice. The role of *OsbHLH024* was investigated in rice under salt stress. CRISPR/Cas9 was applied to edit the *OsbHLH024* gene, and the resulting mutants showed an increase in shoot weight and chlorophyll contents. *OsbHLH024* (Table 2) mutants also showed a considerable improvement in antioxidant activity and a reduced level of ROS (Alam et al., 2022). The potential genetic editing targets have been explored in rice to apply CRISPR/Cas9 for salinity tolerance. The CRISPR/Cas9 mediated knockout of *OsmiR535* showed enhanced resistance to NaCl in mutants (Yue et al., 2020). The key enzyme involved in the xanthophyll cycle is violaxanthin de-epoxidase (VDE). CRISPR/Cas9 mediated gene editing of *OsVDE* showed that overexpression of the gene in transgenic plants did not increase the salinity tolerance in rice, and transgenic plants had no difference from wild type (Wang et al., 2022). Studies have shown that CRISPR/Cas9 mediated circle RNA loci (circRNA) deletion can increase salinity tolerance in rice. CRISPR/Cas9 mediated mutagenesis generated the null mutants for four circRNA loci. Each of the cirRNAloci can be edited at 10% or higher efficiency in both transgenic T_0_ or protoplast. Analysis of mutants revealed that participation of circRNA in salinity response during seedling stage mainly in the *Os05circ02465* increased salinity tolerance (Zhou et al., 2021). The genes involved in sodium homeostasis are key target for CRISPR/Cas9 to mediate plant growth under salinity stress. Wei et al. (2021) demonstrated the targeted editing of clock component *OsPRR73* (Figure 3) which confer salinity tolerance in rice by mediating sodium homeostasis. The CRISPR/Cas9 mediated mutants of *OsPRR73* showed significant improvement in salinity tolerance by reducing cellular Na^+^ accumulation (Wei et al., 2021). ABA is main stress-responsive hormone which controls the germination and growth of seedlings in rice. Knockout of *OsNAC45* genes revealed that transgenic lines showed improvement in germination and growth percentage (Zhang et al., 2020).

**Table 2:** Key applications of CRISPR/Cas9 in the knockout of the salt-tolerant genes in rice.

Gene/protein/enzyme	Trait	Tool	Delivery method	References
*OsVDE*	Dwarfism	CRISPR/Cas9	*Agrobacterium-*mediated transformation	Wang et al., 2022
*BEARI*	Regulated the expression of salt responsive genes, and ions transport	CRISPR/Cas9	*Agrobacterium-*mediated transformation	Teng et al., 2022
*OsbHLH024*	Increase in shoot weight and chlorophyll contents	CRISPR/Cas9	*Agrobacterium tumefaciens* EHA105 strain	Alam et al., 2022
*OsRR22*	Increased plant height and total fresh weight	CRISPR/Cas9	*Agrobacterium-*mediated transformation	Han et al., 2022
*OsMIR408*	Increased salinity tolerance during the seedling stage	CRISPR/Cas9	*Agrobacterium* EHA105	Zhou et al., 2021
*OsPRR73*	Reduced cellular accumulation of Na^+^	CRISPR/Cas9	*Agrobacterium-*mediated transformation	Wei et al., 2021
*OsEC1*	Early heading date	CRISPR/Cas9	*A. tumefaciens* EHA105	Wang et al., 2021
*OsNAC3*	Regulates ABA response	CRISPR/Cas9	*Agrobacterium-*mediated transformation	Zhang et al., 2021
*OsmiR535*	Improved resistance to NaCl	CRISPR/Cas9	*A. tumefaciens* strain EHA105	Yue et al., 2020
*OsNAC45*	Regulates germination and seedling growth	CRISPR/Cas9	*Agrobacterium-*mediated transformation	Zhang et al., 2020
*OsMPT3*	Modulates ATP synthesis and reduces the accumulation of salt ions	CRISPR/Cas9	*Agrobacterium-*mediated transformation	Huang et al., 2020
*BG3*	Increased grain size and CK transport	CRISPR/Cas9	*A. tumefaciens*	Yin et al., 2020
*OsDST*	Higher grain yield	CRISPR/Cas9	*A. tumefaciens* strain EHA105	Santosh Kumar et al., 2020
*OsOTS1*	Zero off-target effects	CRISPR/Cas9	*Agrobacterium-*mediated transformation	Zhang et al., 2019b
*OsNAC041*	Increased plant height in mutants	CRISPR/Cas9	*A. tumefaciens* strain EHA105	Bo et al., 2019
*OsBBS1*	Early leaf senescence	CRISPR/Cas9	*Agrobacterium* strain EHA105	Zeng et al., 2018

Certain genes are the negative controllers of salinity tolerance in rice. The generation of *osRR9*, and *osRR10* double mutants demonstrated that these could affect the expression of multiple genes and have functionally differentiated to control stress response (Wang et al., 2019). The circadian clock plays a key role in the salinity stress response in rice. CRISPR/Cas9 mutated the *OsEC1* to investigate its role under salinity stress. The *osgi-101* mutant created by CRISPR/Cas9 is salt tolerant and showed an early heading date under salinity stress (Wang et al., 2021). Likewise, *OsSPL10* also plays a key role in salinity tolerance in rice. It is considered a negative controller of salinity tolerance in rice which is shown by its involvement in initiation rather than the elongation of trichomes (Lan et al., 2019). The rice gene *OsMPT3* is an important osmotic regulatory factor that enhances salinity tolerance. CRISPR/Cas9 mediated mutagenesis of *OsMPT3* revealed that this gene modulates adenosine triphosphate (ATP) synthesis, reduces ions accumulation, and increases salinity tolerance (Huang et al., 2020). Members of TFs families play a key role in response to salinity stress in rice. Zhang et al. (2021) applied CRISPR/Cas9 tool to edit the *OsNAC3* to test the hypothesis about its role in ABA response and salinity tolerance in rice. Overexpression of *OsNAC3* (Table 2) reduced salts accumulation and increased salinity tolerance in rice (Zhang et al., 2021). Another member *BEAR1* of the TFs family bHLH, acts as a positive regulator of salinity tolerance in rice. CRISPR/Cas9 mediated mutagenesis of *BEAR1* exhibited significant changes in rice under salt stress. *BEAR1* expression is induced by salinity stress and is dominantly expressed in root, seedling stage, and spikelet. *BEARI* enhanced salinity tolerance in rice by regulating the expression of salt-responsive genes and ions transport (Teng et al., 2022). Studies have shown that salt-sensitive TFs could be edited using CRISPR to alter their function under salinity stress. CRISPR/cas9 mediated editing of *OsNAC041* (Table 2) showed increased tolerance to salinity stress. *OsNAC041* mutants had higher plant height than wild type. These findings suggested the potential application of TFs and CRISPR/Cas9 in rice resistance breeding (Bo et al., 2019).

In another study, the *OsBBS1* gene (Table 2), which was involved in early leaf senescence and salt sensitivity, was cloned and a guanine insertion was identified in the first exon of *LOC_Os03g24930*. CRISPR/Cas9 tool kit was applied to edit the gene, and *LOC_Os03g24930* mutants were generated, showing early leaf senescence and confirming that *LOC_Os03g24930* was *OsBBS1* gene. The gene *OsBBS1* was expressed in all tissues and found in the main region of the vein in mature leaves (Zeng et al., 2018). Yin et al. (2020) successfully created the mutant *BG3* gene using the CRISPR/Cas9 tool to enhance salt tolerance in rice. *BG3* is involved in the synthesis of purine permease and is responsible for the transport of cytokinin hormone. Results showed that *BG3* mutants were hypersensitive to salinity stress and mutants with overexpression of *BG3* exhibited improved tolerance to salinity stress and large grains size (Yin et al., 2020). Although many success stories have been published that exhibited the critical role of CRISPR/Cas9 in rice against salinity stress, they did not cover all aspects of salinity stress and its complex nature. There is still a need to use the latest Cas9 variants like Cas10, 12, and 13 which have higher efficiency than Cas9. In future the use of prime editing (PE) and base editing (BE) system will ensure the incredible success of gene editing for salinity tolerance in rice. There is limited information about CRISPR/Cas9 mediated editing of TFs which have a key role in controlling rice response to abiotic stresses. Knockout of salt-sensitive TFs will enhance the salinity tolerance and increase our understanding of the complex genetics of salt stress. It would be better to explore the wild relatives of rice to increase the genetic diversity in our rice pool to counter the adverse effects of climatic changes.

## Transcription factor analysis

**Figure 4:**
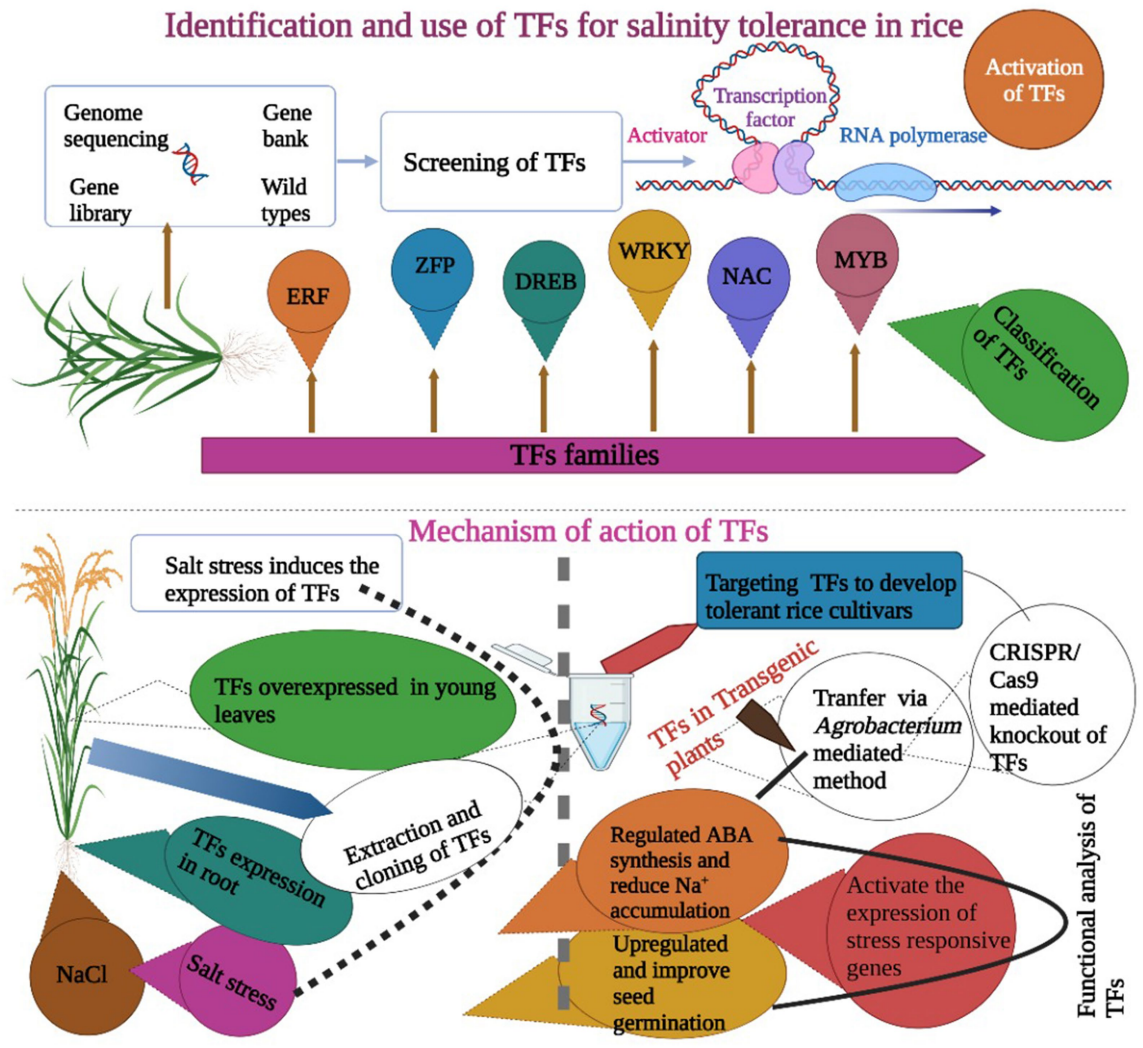
Role of TFs in the development of salt-tolerant rice cultivars. It is obvious that the expression of TFs is induced by salt stress. TFs are overexpressed in leaves and roots and increase salt tolerance *via* ABA synthesis regulation, improving seed germination and increasing the activity of antioxidant enzymes. These TFs are transformed into rice cultivars *via* genetic engineering or targeted using the CRISPR/Cas9 gene-editing tool.

TFs play a key role in the development of stress tolerance in rice. The expression of TFs is often initiated by stress factors (Krishnamurthy et al., 2020a). There is plenty of evidence that shows that TFs are involved in improving salinity tolerance in rice. TFs are proteins that regulate gene expression during stress situations and are divided into many families like NAC, DREB, and bZIP (Figure 4). Although various TFs have been identified and targeted to develop salinity tolerance in rice, but they are not fully characterized and understood. The NAC TF, *OsNAC2* had a key role in salinity tolerance in rice *via* an ABA-mediated pathway. Rice genotypes were evaluated under salt stress (NaCl), and results showed ABA synthesis genes *OsNCED1* and *OsNCED3* in the lines with overexpression of *mOsNAC2* and expression level of stress-responsive genes were higher in these plants (Jiang et al., 2019). TFs are often expressed in roots when exposed to salinity stress. The TF, *OsMADS25* expressed in roots and improved salinity tolerance in rice. Its expression enhanced the primary root length and root density under salinity stress. This potential TF can be used to improve salinity tolerance *via* an ABA-mediated pathway (Xu et al., 2018). Rice plants were exposed to NaCl stress, and overexpression of *AtMYC2* and *AtbHLH122* improved salinity tolerance *via* ABA-mediated pathways (Krishnamurthy et al., 2019).

The mads-box TF, *OsMADS25* plays a key role in root development in rice. MADS gene in some species played a key role in salinity tolerance, but functional features of *OsMADS25* (Table 3) are still not understood. However, its overexpression in rice enhanced salinity tolerance in rice as compared to wild type by increasing proline content and lowering MDA content (Wu et al., 2020). The saltol QTL TF, *OsGATA8* significantly improved salinity tolerance in rice by increasing seed size, photosynthesis, and reducing Na^+^/K^+^ contents. *OsGATA8* (Table 3) induced these effects by regulating ROS critical gene expression and scavenging under salinity stress (Nutan et al., 2020). It is clear from the above studies that the expression of TFs is induced by salinity stress and can be increased or decreased depending on the duration of salinity stress. Trihelix TF plays a key role in plant morphological response to salinity stress. *sGTγ-2* affects the nucleus and is expressed in roots, stems, and rice seeds. Its expression was induced by salinity stress. Results showed that rice seed germination rate, seedling growth, and seedling survival rate was enhanced under salinity stress in the lines with overexpression of *sGTγ-2* (Table 3) lines (Liu et al., 2020c). MBA TF is also involved in the improvement of salinity tolerance in rice. Tang et al. (2019) cloned and characterized the *OsMYB6* gene of MYB TF induced by salinity stress and located in the nucleus. Overexpression of *OsMYB6* in plants increased tolerance to salinity stress by increasing proline contents and higher activities of antioxidant enzymes. It reduced the MDA contents in transgenic plants compared to wild type (Tang et al., 2019).

**Table 3:** List of key TFs involved in salinity tolerance in rice.

TFs/genes	Function	References
*SiMYB19*	Increased grain yield, shoot length, and regulated ABA synthesis	Xu et al., 2022
*GmNAC20*	Reduced MDA content and reduced electrolyte leakage with enhanced activity of antioxidants enzymes	Yarra and Wei, 2021
*OsWRKY87*	Worked as a transcriptional originator	Yan et al., 2021
*OsERF19*	*OsERF19* improved the transcription rate of *OsOTS1* and *OsNCED5* by targeting their promoters’ region	Huang et al., 2021
*OsMADS57*	Improved germination rate, growth rate and longer root length in rice plants	Wu et al., 2021
*OsERF106MZ*	Negative controller of salinity tolerance	Chen et al., 2021a
*OsNAC2*	Increased the expression level of ABA gene, *OsNCED1* and *OsNCED3*	Jiang et al., 2019
*OsMADS25*	Increased proline content and reduced MDA accumulation	Wu et al., 2020
*OsGATA8*	Increased seed size, regulated genes expression and scavenging of ROS	Nutan et al., 2020
*sGTγ-2*	Improved seed germination rate, seedling growth and survival rate	Liu et al., 2020c
*AtMYC2, AtbHLH122*	Regulated ABA mediated pathways	Krishnamurthy et al., 2019
*OsMYB6*	Increased proline content and antioxidant enzymes activities	Tang et al., 2019
*OsZFP213*	Overexpression of *OsZFP213* increased expression of stress related genes, and catalytic activity of enzymes like, CAT, SOD, and APX	Zhang et al., 2018
*IDSI*	Repressed the activity of *LEA1* and *SOS1 genes*	Cheng et al., 2018
*OsMADS25*	Enhanced the primary root length and root density under salinity stress	Xu et al., 2018
*OsPCF2*	Improved salinity tolerance by differential expression	Almeida et al., 2017
*OsNF-YC13*	Increased proline contents, chlorophyl contents, and activity of antioxidants enzymes	Manimaran et al., 2017
*AmRosea1*	Involved in stress signal transduction, hormonal signal pathway, and ions homeostasis	Dou et al., 2016
*OsHKT1;1/OsMYBc*	Reduced Na^+^ accumulation and salinity stress	Wang et al., 2015
*OsbZIP71*	Improved salinity tolerance in rice by activating stress responsive genes	Liu et al., 2014
*OsHsfC1b*	Enhanced plant growth	Schmidt et al., 2012
*TaSRG*	*TaSRG* overexpression enhanced salinity tolerance in rice	He et al., 2011

Zinc finger proteins (ZFP) are the largest group of TFs and play a significant role in salinity tolerance in rice. A ZFP TF, *OsZFP213* reportedly plays a key role in alleviating salt stress in rice through its transactivation activity. Transgenic plants exhibited enhanced tolerance to salinity stress as compared to wild plants. Results revealed that *OsZFP213* leads to increased expression of stress-related genes and catalytic activity of enzymes like catalase (CAT), superoxide dismutase (SOD), and ascorbate peroxidase (APX; Zhang et al., 2018). Identifying stress-responsive pathways is critical to developing salinity tolerance in rice. Cheng et al. (2018) reported the detection of ethylene-responsive TF, INDETERMINATE SPIKELET1(IDSI) and its role in the regulation of rice salt tolerance. The genetic screening revealed the involvement of IDSI in transcriptional repression activity and its role in negatively controlling salinity tolerance. IDSI repressed the activity of *LEA1* and *SOS1* genes (Cheng et al., 2018).

Another NAC TF, *GmNAC20*, was transformed into rice plants to improve salinity tolerance. The integration of *GmNAC20* enhanced chlorophyll content, proline content, and relative water content in T_3_ plants. Moreover, transgenic plants showed lower MDA content and reduced electrocyte leakage with enhanced antioxidant activity under salinity conditions (Yarra and Wei, 2021). As previously discussed, MADS-box TFs have indispensable functions in salinity stress conditions. In a recent study, the role of *OsMADS57* TF was studied under salinity stress conditions. The study showed that NaCl induced the TF expression and it is mainly expressed in leaves and roots. Overexpression of *OsMADS57* improved germination rate, growth rate, and longer root length in rice plants (Wu et al., 2021). One of the most significant TFs classes is ethylene-responsive factors (ERFs) which play a major role in salinity tolerance in rice. The function of ERFs genes is mainly unknown. The gene, *OsERF106*, designated as *OsERF106MZ*, and had negative function in salinity tolerance. Results demonstrated that *OsERF106MZ* was expressed in young flowers, germinating seeds, and roots. Conversely, this gene is the negative controller of salinity tolerance in rice (Chen et al., 2021a). Another ERF is *OsERF19* which had a key role in salt tolerance. *OsERF19* plants exhibited stress tolerance and ABA hypersensitivity compared to wild type. Further analysis revealed that *OsERF19* enhanced the transcription rate of *OsOTS1* and *OsNCED5* by targeting their promoters’ regions (Huang et al., 2021). WRKY TFs family has been studied and characterized for their key role in abiotic stress tolerance. *OsWRKY87* overexpression in transgenic rice plants showed improved tolerance to salinity stress. *OsWRKY87* worked as a transcriptional originator, as proved by yeast one-hybrid assay (Yan et al., 2021). A potential R2R3-MYB TF, *SiMYB19* from foxtail millet is overexpressed mostly in the roots and is brought by salt stress. *SiMYB19* enhances salinity tolerance in rice at seed germination and seedling stage. Transgenic plants with overexpression of *SiMYB19* showed increased grain yield and shoot length. *SiMYB19* regulated the ABA synthesis pathway and increased salinity tolerance (Xu et al., 2022).

The bZIP, a major TFs family, has been studied for salinity tolerance in rice. A previous study showed the characterization of bZIP TF, *OsbZIP71*, which is actively involved in salinity tolerance in rice. Its overexpression is induced by salinity stress. Rice lines with overexpression of *OsbZIP71* exhibited higher salinity tolerance as compared to control conditions (Liu et al., 2014). It is well stated that different TFs of different families expressed under salinity stress, which can be a potential target for a salt breeding program. Almeida et al. (2017) identified five TFs belonging to five families and belonging to the promotor region of the *OsNHX1* gene. These TFs improved salinity tolerance through different expressions in rice plants (Almeida et al., 2017). Because of the diversity of TFs, they can be transferred into another crop to enhance salinity tolerance. A wheat TF, *TaSRG*, significantly affects the salinity tolerance in rice. *TaSRG* encodes a protein that is in the nucleus. Overexpression of *TaSRG* increased salinity tolerance in rice and Arabidopsis (He et al., 2011). Heat shock transcription factors (HSFs) are also overexpressed under salinity stress and reduce the risk of attenuated growth in rice. An HSFs, *OsHsfC1b* in rice overexpressed when plants were exposed to salinity stress. *OsHsfC1b* increased salinity tolerance by increasing plant growth (Schmidt et al., 2012).

Sodium transporters often regulate the transport of sodium and potassium ions out of the cell in rice. A potassium transporter, *OsHKT1;1* reduced Na^+^ accumulation in cells to reduce salinity stress in rice. Mutants with overexpression of *OsHKT1;1* (Table 3) showed hypersensitivity to salinity stress. *OsHKT1;1* was expressed in phloem sap, and its expression is upregulated by salinity stress (Wang et al., 2015). Many studies revealed the role of nuclear factors (NF-F) in salinity tolerance in rice. Manimaran et al. (2017) reported the *OsNF-YC13*, an NF-Y TF whose transcript level was upregulated in tissues by salinity stress treatment. It enhanced the proline contents, chlorophyll contents, and activity of antioxidant enzymes (Manimaran et al., 2017). The detailed overview of TFs analysis of rice showed that these factors played a key role in improving salinity tolerance in rice. However, all TFs are not characterized and analyzed for their role in salinity tolerance in rice. bZIP and WRKY families of TFs are least explored in rice. It is mandatory to increase the expression of TFs in rice by exposing to different salinity stress levels. The exploration of wild relatives of rice could be significant technique to increase the genetic diversity for salinity tolerance in rice. The identification of novel TFs would be a potential target for CRISPR/Cas9 and genetic engineering to develop salt-tolerant rice genotypes suitable to grown on saline soils to sustain growth and yield.

## Applications of genetic engineering to breed salt-tolerant rice

Genetic engineering has been a potent tool for developing salt-resistant rice cultivars by introducing selected genes into elite cultivars (Ali et al., 2021; Chapagain et al., 2021). The role of ion transporters, antioxidants, and TFs is well known, so they have been transferred to engineer salinity tolerance in rice (Ganie, 2020). However, numerous studies have been published which described the exploitation of different methods used to develop salinity tolerance in rice. However, genetic engineering is one of the most feasible approaches. MicroRNAs (miRNAs) are well-known as key gene regulators and have an important contribution to stress tolerance. Fan et al. (2022) successfully revealed the role of miRNAs in salt tolerance. They have selected 2 out of 18 lines with higher expression of miRNAs for salt breeding. RNA-seq analysis uncovered the 1980 genes with different expressions in transgenic lines in rice (Fan et al., 2022). The functional characterization of genes is critical in developing salt-tolerant transgenic lines in rice. The biological role of the *OSRIP18* gene was studied in the rice using genetic engineering technique. Eleven transgenic lines were developed, showing an improved tolerance to salinity stress (Jiang et al., 2012). Not only genes but many TFs have also been cloned and transferred to develop transgenic rice cultivars resistant to salt stress. *ATAF1* member of the NAC family was cloned and transferred using the *Agrobacterium*-mediated transformation method to develop transgenic rice lines. Transgenic rice lines showed increased tolerance to salinity stress with insensitivity to ABA (Liu et al., 2016). Xu et al. (2014) reported the role of the *OsMsr9* gene in salt tolerance in rice. *OsMsr9* overexpression in transgenic rice plants showed a higher survival rate and enhanced root and shoot growth under salinity stress (Xu et al., 2014). Identifying new sources of salt tolerance is important for a successful breeding program.

*NaMnSOD* (Table 4) was transformed to generate transgenic rice lines with the improved character of salinity tolerance. As a result, transgenic lines showed reduced H_2_O_2_ contents and a higher photosynthesis rate (Chen et al., 2013). Likewise, overexpression of *OsACBP4* protects rice plants from salinity stress via regulation of lipid metabolism (Guo et al., 2021). Different ribosomal proteins also play a key role in modulating stress tolerance in rice. The ribosomal protein *RPL6* (Table 4) modulated salinity tolerance in transgenic rice under 200 mM NaCl. Rice transgenic plants exhibited greater phenotypic response, higher chlorophyll contents, proline contents, and yield than wild type (Moin et al., 2021). Genetic engineering resulted in the transformation of genes that reduced salinity tolerance in rice, as revealed earlier by (Zhang et al. 2022), who showed that *OsSCL30* reduced tolerance to salt stress in rice.

**Table 4:** Engineered genes for salinity tolerance in rice.

Transgene	Function	References
*OsABA8ox1*-kd	Reduced ROS contents and Na^+^/K^+^ ratio in transgenic seedlings	Liu et al., 2022c
*OsCYP2*	Enhanced salinity tolerance by increasing growth under stress	Liu et al., 2022a
*OsCYP2-P*	Reduction in toxic cellular ions accumulation	Roy et al., 2022
*OsSOS2*	Maintain ions homeostasis	Kumar et al., 2022
*OsSKL2*	Increased the activity of antioxidant enzymes and reduced electrolyte leakage	Jiang et al., 2022
*OsSCL30*	Negative controller of salinity tolerance	Zhang et al., 2022
*RPL6*	Increased chlorophyll and proline contents and seed yield	Moin et al., 2021
*OsACBP4*	Improved lipid metabolism	Guo et al., 2021
*ATAF1*	Increased insensitivity to ABA	Liu et al., 2016
*OsMsr9*	The increased survival rate, and root growth	Xu et al., 2014
*NaMnSOD*	Reduced H_2_O_2_ content and increased photosynthesis rate	Chen et al., 2013
*OSRIP18*	Involved in up-regulation of stress-dependent or independent genes	Jiang et al., 2012

*OsSKL2* gene was characterized for its role in salinity tolerance in rice. *Agrobacterium*-mediated transformation method was used to develop transgenic lines of rice. *OsSKL2* overexpression increased tolerance to salinity stress by increasing the activity of antioxidant enzymes and reducing electrolyte leakage (Jiang et al., 2022). A homolog *OsSOS2* of SOS2 in rice improved salinity tolerance. Transgenic plants overexpressed *OsSOS2* showed enhanced salinity tolerance by maintaining favorable ions homeostasis, indicating its potential biotechnological application in improving salt breeding programs (Kumar et al., 2022). In another study, Roy et al. (2022) studied the expression of the *OsCYP2-P* (Table 4) gene in salinity stress response in rice. Transgenic plants showed a reduction in toxic cellular ions accumulation and improved ions homeostasis (Roy et al., 2022).

In the same way, the *OsCYP2* gene that constitutes the salinity tolerance characters was transformed into rice cultivars which increased salinity tolerance in transgenic rice (Liu et al., 2022a). These recent studies have increased our understanding of improving salinity tolerance in rice *via* applying genetic engineering. The key abiotic catalytic gene, *OsABA8ox1*-kd, improved salinity tolerance in rice. *OsABA8ox1*-kd overexpressing seedlings had a higher level of ABA, reduced ROS contents, and Na^+^/K^+^ ratio under salinity stress compared to non-transgenic plants (Liu et al., 2022c). Genetic engineering has achieved a landmark position in molecular breeding because of its role in developing several tolerant crop cultivars (Li et al., 2022a; Liu et al., 2022b).

Although detailed research reports have been published about salinity tolerance in rice, all TFs and genes are not characterized for their potential applications in salt breeding programs. Screening wild germplasm is a feasible way to identify the gene of interest. Haplotype plants have demonstrated their ability to thrive under extreme salinity stress conditions, and they are the best source of salt-tolerant genes. Landraces are a rich source of stable genes for yield improvement under salinity stress. Meanwhile, the efficiency of the *Agrobacterium* vector needs to be improved for successful transformation. In the future, the modified genetic transformation methods would help rapidly increase the salt breeding programs to cope with environmental fluctuations. Conservation of rice genetic diversity is critical for genetic engineering to safeguard the yield in extreme environmental conditions that gradually reduce the crop yield and quality and create an imbalance in the food security system.

## Allele mining

QTL/genes regulating salinity tolerance show genetic variation among diverse genotypes. Genetic variation is a prerequisite to identifying the potential candidate allele for salt breeding (Chen et al., 2021b). A potential allele of salt inducible rice gene (Sal T) was discovered from various cultivated and wild rice genotypes (Ganie et al., 2014). Meanwhile, allelic diversity in the coding sequence (CDS) for key salt-tolerant genes, calcium-dependent protein kinase17 (*OsCPK17*), and *OsHKT1;5* were identified in 392 rice accessions (Negrão et al., 2013). Genetic analysis exhibited the six ecotypic variants of gene *OsKHT2:1* were detected with novel HKT isoform, *No-OsHKT2;2/1* in Nona Bokra, added to salinity tolerance in rice (Oomen et al., 2012). The Aromatic allele of gene *OsHKT1;5* showed the lowest Na^+^ concentration in rice leaves (Platten et al., 2013). In a recent study, the haplotypes of *HKT1;5NB* (*OsHKT1;5* identified in Nona Bokra) signifying the “His” amino acids demonstrated a lower concentration of Na^+^ in rice straws (Yang et al., 2018). The conducive information provided by two *OsHKT1:5* alleles is useful when choosing superior genotypes. Likewise, resequencing of eight KHT genes of 103 rice accessions was completed, revealing the novel salt-tolerant alleles of genes *HKT1;5* and *HKT2;3* (Mishra et al., 2016). Current findings about high-throughput genotyping approaches have improved the influence of allele discovery. 3,000 rice genomes project (3 K RGP; 3, 3000 Rice Genomic Project, 2014) was started for genomic sequencing of 3,000 rice accessions representing inherited and significant assortment. During the past years, this germplasm has been widely used in mapping of QTL and alleles mining for varied characters comprising salinity tolerance in rice (Campbell et al., 2017; Liu et al., 2019). The documentation of promising alleles of diverse genes or QTL for salt tolerance will assist to find more useful evidence for salt breeding in rice (Chen et al., 2021b).

## Conclusion and recommendations

The world population continuously surges and puts unbearable pressure on available natural resources. Salinity stress is considered a major issue due to its detrimental effects on agricultural crop productivity and sustainability. If not properly managed, salinity stress can eliminate rice yield worldwide. Salinity stress has shrunk the rice yield and quality to above a certain salinity threshold. There are no clear estimates of the percentage of the land area damaged by salinity stress; however, according to a rough estimate 412 million hectares of land have been affected worldwide. These threats forced rice breeders and researchers to find the solution to counter severe salinity stress issues. Rice responds to salt stress by adopting morphological, physiological, and biochemical mechanisms. The plant maintains ions homeostasis, reduces Na^+^/Cl^−^ ratio, and scavenges ROS in the cell by various mechanisms. The genetic mechanism of salinity stress tolerance is complex and needs further studies. For decades breeders have been engaged in stabilizing the agricultural food security system by developing salt-tolerant rice cultivars. Rice is a major staple food for around 50% of the world population, mainly in Asian countries. The earlier era of conventional breeding embraced the challenges of salt breeding and developed several salt-tolerant rice cultivars, which resulted in higher yields on salt-affected areas.

Conventional breeding methods have limited use in the modern era of crop genetics and breeding. Molecular breeding approaches have enhanced the speed of salt breeding and ensured rice production in extreme environmental conditions. QTL mapping is a reliable approach to identifying the potential genomic regions controlling salt tolerance in rice and their use in marker-assisted selection (MAS). The major QTL involved in salinity tolerance in rice have been briefly discussed in this review; however, not all of them are cloned and transformed via QTL pyramiding. Hence, these QTL must be cloned and transformed to develop salt-tolerant rice cultivars. Genetic engineering has increased the rice capability to yield higher under salt stress, and today hundreds of transgenic cultivars are grown commercially. It would be better to screen the wild germplasm and characterize the gene used in future breeding programs.

Meanwhile, many TFs families have not been fully analyzed for their role in salt tolerance. TFs have been critically analyzed regarding their role in salt tolerance, and most of them showed higher expression under salt stress. CRISPR/Cas9 for targeted gene editing of salt-tolerant genes has gained much attention in the molecular breeding field. Hundreds of mutants created by CRISPR/Cas9 showed increased salt tolerance under stress conditions. We strongly recommend using novel editing systems like base editing (BE) and prime editing (PE) to increase gene-editing efficiency. CRISPR/Cas9 use would enhance genetic diversity for salt breeding programs. These combined efforts would ensure food security and meet the food requirements of the rapidly growing human population.

## Author contributions

AR conceptualized and prepared the manuscript. HL provided technical assistance. MN, AM, MUH, ANS, FH, SA, reviewed the manuscript. SFAG, YM, SHQ provided technical assistance. ZW reviewed the manuscript, supervised the study, and provided funding. All authors contributed to the article and approved the submitted version.

## Funding

The research was supported by the National Natural Science Foundation of China (31760350 and 71963020), the National Key Research and Development Program of China (2018YFD0301102), the Key Research and Development Program of Jiangxi Province (20171ACF60018 and 20192ACB60003), Natural Science Foundation of Jiangxi (20202BABL205020), the Jiangxi Agriculture Research System (JXARS-18), and Training Program for Academic and Technical Leaders in Major Discipline in Jiangxi Province (20204BCJL22044).

## Conflict of interest

The authors declare that the research was conducted in the absence of any commercial or financial relationships that could be construed as a potential conflict of interest.

## Publisher’s note

All claims expressed in this article are solely those of the authors and do not necessarily represent those of their affiliated organizations, or those of the publisher, the editors and the reviewers. Any product that may be evaluated in this article, or claim that may be made by its manufacturer, is not guaranteed or endorsed by the publisher.

## References

[ref1] 3000 Rice Genomic Project (2014). The 3,000 rice genomes project. Gigascience 3:7. doi: 10.1186/2047-217X-3-7 24872877 PMC4035669

[ref2] Abdallah M.-S. Abdelgawad Z. El-Bassiouny H. (2016). Alleviation of the adverse effects of salinity stress using trehalose in two rice varieties. South Afr. J. Bot. 103, 275–282. doi: 10.1016/j.sajb.2015.09.019

[ref3] Alam M. N. U. Jewel G. N. A. Azim T. Seraj Z. I. (2021). Novel QTLs for salinity tolerance revealed by genome-wide association studies of biomass, chlorophyll and tissue ion content in 176 rice landraces from Bangladesh. PLoS One 16:e0259456. doi: 10.1371/journal.pone.0259456, PMID: 34739483 PMC8570475

[ref4] Alam M. S. Kong J. Tao R. Ahmed T. Alamin M. Alotaibi S. S.. (2022). CRISPR/Cas9 mediated knockout of the *OsbHLH024* transcription factor improves salt stress resistance in rice (*Oryza sativa* L.). Plan. Theory 11:1184. 10.3390/plants11091184 PMC9101608 35567185

[ref5] Ali F. Chen W. Fiaz S. Wang Y. Wei X. Xie L.. (2022). QTL mapping for grain appearance quality traits using doubled haploid population of rice under different environments. Pak. J. Bot. 54, 1265–1275.

[ref6] Ali U. Shar T. Ahmad R. Khatoon M. Khaskheli M. A. Laghari A. H.. (2021). Salinity stress–a threat to rice production breeding strategies to develop salinity tolerance in plants. Meh. J. Sci. Tech. 1, 13–17.

[ref7] Almeida D. M. Gregorio G. B. Oliveira M. M. Saibo N. J. (2017). Five novel transcription factors as potential regulators of *OsNHX1* gene expression in a salt tolerant rice genotype. Plant Mol. Biol. 93, 61–77. doi: 10.1007/s11103-016-0547-7, PMID: 27766460

[ref8] Amin U. Biswas S. Elias S. M. Razzaque S. Haque T. Malo R.. (2016). Enhanced salt tolerance conferred by the complete 2.3 kb cDNA of the rice vacuolar Na+/H+ antiporter gene compared to 1.9 kb coding region with 5′ UTR in transgenic lines of rice. Front. Plant Sci. 7:14. doi: 10.3389/fpls.2016.00014, PMID: 26834778 PMC4724728

[ref9] Amirjani M. R. (2011). Effect of salinity stress on growth, sugar content, pigments and enzyme activity of rice. Int. J. Bot. 7, 73–81. doi: 10.3923/ijb.2011.73.81

[ref10] Arifuddin M. Musa Y. Farid M. Anshori M. Nasaruddin N. Nur A.. (2021). Rice screening with hydroponic deep-flow technique under salinity stress. SABRAO J. Breed. Gen. 53, 435–446.

[ref11] Bizimana J. B. Luzi-Kihupi A. Murori R. W. Singh R. (2017). Identification of quantitative trait loci for salinity tolerance in rice (*Oryza sativa* L.) using IR29/Hasawi mapping population. J. Gen. 96, 571–582. doi: 10.1007/s12041-017-0803-x, PMID: 28947705

[ref12] Bo W. Zhaohui Z. Huanhuan Z. Xia W. Binglin L. Lijia Y.. (2019). Targeted mutagenesis of NAC transcription factor gene, *OsNAC041*, leading to salt sensitivity in rice. Rice Sci. 26, 98–108. doi: 10.1016/j.rsci.2018.12.005

[ref13] Bundó M. Martín-Cardoso H. Gómez-Ariza J. Pesenti M. Castillo L. Frouin J.. (2022). Integrative approach for precise genotyping and transcriptomics of salt tolerant introgression rice lines. Front. Plant Sci. 12:3217. 10.3389/fpls.2021.797141 PMC8813771 35126422

[ref14] Campbell M. T. Bandillo N. Al Shiblawi F. R. A. Sharma S. Liu K. Du Q.. (2017). Allelic variants of OsHKT1; 1 underlie the divergence between indica and japonica subspecies of rice (*Oryza sativa*) for root sodium content. PLoS Genet. 13:e1006823. doi: 10.1371/journal.pgen.1006823, PMID: 28582424 PMC5476289

[ref15] Channa G. S. Mahar A. R. Memon A. H. Bhagat M. A. Saand M. A. Mirbahar A. A.. (2021). Effect of salinity on emergence and early growth stages of aromatic and non-aromatic Rice (*Oryza sativa* L.) genotypes. Biol. Sci.-PJSIR 64, 64–74. doi: 10.52763/PJSIR.BIOL.SCI.64.1.2021.64.74

[ref16] Chapagain S. Singh L. Garcia R. Pruthi R. Concepcion J. Coronejo S.. (2021). “Molecular breeding for improving salinity tolerance in rice: recent progress and future prospects,” in Molecular Breeding for Rice Abiotic Stress Tolerance and Nutritional Quality. eds. A. H. Mohammad, H. Anwar, H. Lutful, M. I. Khandakar, K. Arvind and H. Robert (John Wiley & Sons Ltd.), 26–52.

[ref17] Chen H.-C. Chien T.-C. Chen T.-Y. Chiang M.-H. Lai M.-H. Chang M.-C. (2021a). Overexpression of a novel ERF-X-type transcription factor, *OsERF106MZ*, reduces shoot growth and tolerance to salinity stress in rice. Rice 14, 1–18. doi: 10.1186/s12284-021-00525-5 34542722 PMC8452809

[ref18] Chen Z. Pan Y. An L. Yang W. Xu L. Zhu C. (2013). Heterologous expression of a halophilic archaeon manganese superoxide dismutase enhances salt tolerance in transgenic rice. Russ. J. Plant Physiol. 60, 359–366. doi: 10.1134/S1021443713030059

[ref19] Chen T. Shabala S. Niu Y. Chen Z.-H. Shabala L. Meinke H.. (2021b). Molecular mechanisms of salinity tolerance in rice. Crop J. 9, 506–520. doi: 10.1016/j.cj.2021.03.005

[ref20] Chen T. Zhu Y. Chen K. Shen C. Zhao X. Shabala S.. (2020). Identification of new QTL for salt tolerance from rice variety Pokkali. J. Agron. Crop Sci. 206, 202–213. doi: 10.1111/jac.12387

[ref21] Cheng X. Zhang S. Tao W. Zhang X. Liu J. Sun J.. (2018). INDETERMINATE SPIKELET1 recruits histone deacetylase and a transcriptional repression complex to regulate rice salt tolerance. Plant Physiol. 178, 824–837. doi: 10.1104/pp.18.00324, PMID: 30061119 PMC6181036

[ref22] Chinnusamy V. Jagendorf A. Zhu J. K. (2005). Understanding and improving salt tolerance in plants. Crop Sci. 45, 437–448. doi: 10.2135/cropsci2005.0437, PMID: 35730915

[ref23] Dahanayaka B. Gimhani D. Kottearachchi N. Samarasighe W. (2017). QTL mapping for salinity tolerance using an elite rice (*Oryza sativa*) breeding population. SABRAO J. Breed. Genet. 49, 123–134.

[ref24] Dai L. Li P. Li Q. Leng Y. Zeng D. Qian Q. (2022). Integrated multi-omics perspective to strengthen the understanding of salt tolerance in rice. Int. J. M. Sci. 23:5236. doi: 10.3390/ijms23095236, PMID: 35563627 PMC9105537

[ref25] De Leon T. B. Linscombe S. Subudhi P. K. (2017). Identification and validation of QTLs for seedling salinity tolerance in introgression lines of a salt tolerant rice landrace ‘Pokkali’. PLoS One 12:e0175361. doi: 10.1371/journal.pone.0178911, PMID: 28388633 PMC5384751

[ref26] de Ocampo M. P. Thomson M. J. Mitsuya S. Yamauchi A. Ismail A. M. (2022). QTL mapping under salt stress in rice using a Kalarata–Azucena population. Euphytica 218, 1–15. doi: 10.1007/s10681-022-03026-8 PMC9427886 36060537

[ref27] Diédhiou C. Golldack D. (2006). Salt-dependent regulation of chloride channel transcripts in rice. Plant Sci. 170, 793–800. doi: 10.1016/j.plantsci.2005.11.014, PMID: 12602892

[ref28] Dou M. Fan S. Yang S. Huang R. Yu H. Feng X. (2016). Overexpression of *AmRosea1* gene confers drought and salt tolerance in rice. Int. J. Mol. Sci. 18:2. doi: 10.3390/ijms18010002 PMC5297637 28025485

[ref29] Fan X. Jiang H. Meng L. Chen J. (2021). Gene mapping, cloning and association analysis for salt tolerance in Rice. Int. J. Mol. Sci. 22:11674. doi: 10.3390/ijms222111674, PMID: 34769104 PMC8583862

[ref30] Fan Y. Zhang F. Xie J. (2022). Overexpression of miR5505 enhanced drought and salt resistance in rice (Orayza sativa). *bioRxiv* doi: 10.1101/2022.01.13.476146 [Epub ahead of preprint].

[ref31] Ganie S. A. (2020). RNA chaperones: potential candidates for engineering salt tolerance in rice. Crop Sci. 60, 530–540. doi: 10.1002/csc2.20134

[ref32] Ganie S. A. Karmakar J. Roychowdhury R. Mondal T. K. Dey N. (2014). Assessment of genetic diversity in salt-tolerant rice and its wild relatives for ten SSR loci and one allele mining primer of salT gene located on 1st chromosome. Plant Syst. Evolut. 300, 1741–1747. doi: 10.1007/s00606-014-0999-7

[ref33] Guo Z.-H. Pogancev G. Meng W. Du Z.-Y. Liao P. Zhang R.. (2021). The overexpression of rice ACYL-COA-BINDING PROTEIN4 improves salinity tolerance in transgenic rice. Environ. Exp. Bot. 183:104349. doi: 10.1016/j.envexpbot.2020.104349

[ref34] Habila S. Khunpolwattana N. Chantarachot T. Buaboocha T. Comai L. Chadchawan S.. (2022). Salt stress responses and SNP-based phylogenetic analysis of Thai rice cultivars. Plant Gen. 15:e20189. doi: 10.1002/tpg2.20189 PMC12807289 34994516

[ref35] Hakim M. Juraimi A. Hanafi M. Ismail M. Rafii M. Islam M.. (2014). The effect of salinity on growth, ion accumulation and yield of rice varieties. J. Ani. Plant Sci. 24, 874–885.

[ref36] Han X. Chen Z. Li P. Xu H. Liu K. Zha W.. (2022). Development of novel rice germplasm for salt-tolerance at seedling stage using CRISPR-Cas9. Sustainability 14:2621. doi: 10.3390/su14052621

[ref37] Haque M. A. Rafii M. Y. Yusoff M. M. Ali N. S. Yusuff O. Datta D. R.. (2021). Advanced breeding strategies and future perspectives of salinity tolerance in rice. Agronomy 11:1631. doi: 10.3390/agronomy11081631

[ref38] Hasanuzzaman M. Fujita M. Islam M. Ahamed K. Nahar K. (2009). Performance of four irrigated rice varieties under different levels of salinity stress. Int. J. Integrat. Biol. 6, 85–90.

[ref39] He X. Hou X. Shen Y. Huang Z. (2011). *TaSRG*, a wheat transcription factor, significantly affects salt tolerance in transgenic rice and Arabidopsis. FEBS Lett. 585, 1231–1237. doi: 10.1016/j.febslet.2011.03.055, PMID: 21457711

[ref40] Huang S. Ma Z. Hu L. Huang K. Zhang M. Zhang S.. (2021). Involvement of rice transcription factor *OsERF19* in response to ABA and salt stress responses. Plant Physiol. Biochem. 167, 22–30. doi: 10.1016/j.plaphy.2021.07.027, PMID: 34329842

[ref41] Huang S. Xin S. Xie G. Han J. Liu Z. Wang B.. (2020). Mutagenesis reveals that the rice *OsMPT3* gene is an important osmotic regulatory factor. Crop J. 8, 465–479. doi: 10.1016/j.cj.2020.02.001, PMID: 31244876

[ref42] Irakoze W. Prodjinoto H. Nijimbere S. Rufyikiri G. Lutts S. (2020). NaCl and Na2SO4 salinities have different impact on photosynthesis and yield-related parameters in rice (*Oryza sativa* L.). Agronomy 10:864. doi: 10.3390/agronomy10060864

[ref43] Islam M. A. De Bruyn L. L. Warwick N. W. Koech R. (2021). Salinity-affected threshold yield loss: a signal of adaptation tipping points for salinity management of dry season rice cultivation in the coastal areas of Bangladesh. J. Environ. Manag. 288:112413. doi: 10.1016/j.jenvman.2021.112413, PMID: 33845271

[ref44] Jahan N. Zhang Y. Lv Y. Song M. Zhao C. Hu H.. (2020). QTL analysis for rice salinity tolerance and fine mapping of a candidate locus *qSL7* for shoot length under salt stress. Plant Growth Reg. 90, 307–319. doi: 10.1007/s10725-019-00566-3

[ref45] Jiang S.-Y. Bhalla R. Ramamoorthy R. Luan H.-F. Venkatesh P. N. Cai M.. (2012). Over-expression of *OSRIP18* increases drought and salt tolerance in transgenic rice plants. Transgenic Res. 21, 785–795. doi: 10.1007/s11248-011-9568-9, PMID: 22038450

[ref46] Jiang Y. Peng X. Zhang Q. Liu Y. Li A. Cheng B.. (2022). Regulation of drought and salt tolerance by *OsSKL2* and *OsASR1* in rice. Rice. doi: 10.21203/rs.3.rs-1491451/v1 PMC9424430 36036369

[ref47] Jiang D. Zhou L. Chen W. Ye N. Xia J. Zhuang C. (2019). Overexpression of a microRNA-targeted NAC transcription factor improves drought and salt tolerance in rice via ABA-mediated pathways. Rice 12, 1–11. doi: 10.1186/s12284-019-0334-6 31637532 PMC6803609

[ref48] Khan M. Kamal S. Saeed M. Iqbal J. (2016). Quantitative trait locus mapping for salt tolerance at maturity stage in indica rice using replicated F_2_ population. Braz. J. Bot. 39, 641–650. doi: 10.1007/s40415-016-0272-0

[ref49] Kong W. Zhang C. Zhang S. Qiang Y. Zhang Y. Zhong H.. (2021). Uncovering the novel QTLs and candidate genes of salt tolerance in rice with linkage mapping, RTM-GWAS, and RNA-seq. Rice 14, 1–12. doi: 10.1186/s12284-021-00535-3 34778931 PMC8590990

[ref50] Kotula L. Garcia Caparros P. Zörb C. Colmer T. D. Flowers T. J. (2020). Improving crop salt tolerance using transgenic approaches: An update and physiological analysis. Plant Cell Environ. 43, 2932–2956. doi: 10.1111/pce.13865, PMID: 32744336

[ref51] Krishnamurthy S. Pundir P. Warraich A. S. Rathor S. Lokeshkumar B. Singh N. K.. (2020b). Introgressed saltol QTL lines improves the salinity tolerance in rice at seedling stage. Front. Plant Sci. 11:833. doi: 10.3389/fpls.2020.00833 32595689 PMC7300257

[ref52] Krishnamurthy S. Sharma P. Dewan D. Lokeshkumar B. Rathor S. Warraich A.. (2022). Genome wide association study of MAGIC population reveals a novel QTL for salinity and sodicity tolerance in rice. Physiol. Mol. Biol. Plants 28, 819–835. doi: 10.1007/s12298-022-01174-8, PMID: 35592486 PMC9110595

[ref53] Krishnamurthy P. Vishal B. Ho W. J. Lok F. C. J. Lee F. S. M. Kumar P. P. (2020a). Regulation of a cytochrome *P450* gene *CYP94B1* by *WRKY33* transcription factor controls apoplastic barrier formation in roots to confer salt tolerance. Plant Physiol. 184, 2199–2215. doi: 10.1104/pp.20.01054 32928900 PMC7723105

[ref54] Krishnamurthy P. Vishal B. Khoo K. Rajappa S. Loh C.-S. Kumar P. P. (2019). Expression of *AoNHX1* increases salt tolerance of rice and Arabidopsis, and bHLH transcription factors regulate *AtNHX1* and *AtNHX6* in Arabidopsis. Plant Cell Rep. 38, 1299–1315. doi: 10.1007/s00299-019-02450-w, PMID: 31350571

[ref55] Kumar G. Basu S. Singla-Pareek S. L. Pareek A. (2022). Unraveling the contribution of *OsSOS2* in conferring salinity and drought tolerance in a high-yielding rice. Physiol. Plant. 174:e13638. doi: 10.1111/ppl.13638, PMID: 35092312

[ref56] Kumari K. Kumar M. Kim S.-R. Ryu H. Cho Y.-G. (2013). Insights into genomics of salt stress response in rice. Rice 6, 1–15. doi: 10.1186/1939-8433-6-27 24280112 PMC4883734

[ref57] Kumari S. Singh B. Singh S. K. Satya D. Singh S. Tripathy K.. (2021). Exploring novel QTLs among backcross lines for salinity tolerance in rice. Ind. J. Agricul Sci. 91, 1–4.

[ref58] Kurniasih B. Hasanah U. Muflikhah N. (2021). “Rice cultivars responses to different salinity levels at coastal agricultural land of Yogyakarta,” in *IOP Conference Series: Earth and Environmental Science*; November 4–5, 2020; Bristol: IOP Publishing, 012026.

[ref59] Kurusu T. Hamada H. Koyano T. Kuchitsu K. (2012). Intracellular localization and physiological function of a rice Ca2+−permeable channel *OsTPC1*. Plant Sign. Beh. 7, 1428–1430. doi: 10.4161/psb.22086 PMC3548864 22990444

[ref60] Lan T. Zheng Y. Su Z. Yu S. Song H. Zheng X.. (2019). *OsSPL10*, a SBP-box gene, plays a dual role in salt tolerance and trichome formation in rice (*Oryza sativa* L.). G3 9, 4107–4114. doi: 10.1534/g3.119.400700 31611344 PMC6893181

[ref61] Le T. D. Gathignol F. Vu H. T. Nguyen K. L. Tran L. H. Vu H. T. T.. (2021). Genome-wide association mapping of salinity tolerance at the seedling stage in a panel of Vietnamese landraces reveals new valuable QTLs for salinity stress tolerance breeding in rice. Plan. Theory 10:1088. doi: 10.3390/plants10061088 PMC8228224 34071570

[ref62] Lei L. Han Z. Cui B. Yang L. Liu H. Wang J.. (2021). Mapping of a major QTL for salinity tolerance at the bud burst stage in rice (*Oryza sativa* L) using a high-density genetic map. Euphytica 217, 1–8. doi: 10.1007/s10681-021-02901-0

[ref63] Lei L. Zheng H. Bi Y. Yang L. Liu H. Wang J.. (2020). Identification of a major qtl and candidate gene analysis of salt tolerance at the bud burst stage in rice (*Oryza sativa* L.) using QTL-Seq and RNA-Seq. Rice 13, 1–14. doi: 10.1186/s12284-020-00416-1 32778977 PMC7417472

[ref64] Leridon H. (2020). World population outlook: explosion or implosion? Pop. Soc. 573, 1–4.

[ref65] Li X. Guo D. Xue M. Li G. Yan Q. Jiang H.. (2022a). Genome-wide association study of salt tolerance at the seed germination stage in flax (*Linum usitatissimum* L.). Genes 13:486. doi: 10.3390/genes13030486 35328040 PMC8949523

[ref66] Li Y. Zhou J. Li Z. Qiao J. Quan R. Wang J.. (2022b). Salt and ABA response *ERF1* improves seed germination and salt tolerance by repressing ABA signaling in rice. Plant Physiol. 189, 1110–1127. doi: 10.1093/plphys/kiac125 35294556 PMC9157093

[ref67] Linh L. H. Linh T. H. Xuan T. D. Ham L. H. Ismail A. M. Khanh T. D. (2012). Molecular breeding to improve salt tolerance of rice (*Oryza sativa* L.) in the red River Delta of Vietnam. Int. J. Plant Gen. 2012:949038. 10.1155/2012/949038 PMC3544250 23326259

[ref68] Liu C. Chen K. Zhao X. Wang X. Shen C. Zhu Y.. (2019). Identification of genes for salt tolerance and yield-related traits in rice plants grown hydroponically and under saline field conditions by genome-wide association study. Rice 12, 1–13. doi: 10.1186/s12284-019-0349-z 31792643 PMC6889114

[ref69] Liu H. Cui P. Zhang B. Zhu J. Liu C. Li Q. (2022a). Transcription factor myc2-like, binding to ABRE element of *OsCYP2* promoter, enhance salt tolerance in *Oryza sativa*. *Plant Mol. Biol*. Rep. doi: 10.21203/rs.3.rs-1626063/v1 PMC9565382 36240213

[ref70] Liu H. Hu H. Tang K. Rehman M. Du G. Huang Y.. (2022b). Overexpressing hemp salt stress induced transcription factor genes enhances tobacco salt tolerance. Indust. Crops Products 177:114497. doi: 10.1016/j.indcrop.2021.114497

[ref71] Liu C. Mao B. Ou S. Wang W. Liu L. Wu Y.. (2014). *OsbZIP71*, a bZIP transcription factor, confers salinity and drought tolerance in rice. Plant Mol. Biol. 84, 19–36. doi: 10.1007/s11103-013-0115-3, PMID: 23918260

[ref72] Liu M. Pan T. Allakhverdiev S. I. Yu M. Shabala S. (2020a). Crop halophytism: an environmentally sustainable solution for global food security. Tren. Plant Sci. 25, 630–634. doi: 10.1016/j.tplants.2020.04.008 32444156

[ref73] Liu Y. Sun J. Wu Y. (2016). Arabidopsis *ATAF1* enhances the tolerance to salt stress and ABA in transgenic rice. J. Plant Res. 129, 955–962. doi: 10.1007/s10265-016-0833-0, PMID: 27216423

[ref74] Liu X. Wu D. Shan T. Xu S. Qin R. Li H.. (2020c). The trihelix transcription factor *OsGTγ-2* is involved adaption to salt stress in rice. Plant Mol. Biol. 103, 545–560. doi: 10.1007/s11103-020-01010-1 32504260

[ref75] Liu X. Xie X. Zheng C. Wei L. Li X. Jin Y.. (2022c). RNAi-mediated suppression of the abscisic acid catabolism gene *OsABA8ox1* increases abscisic acid content and tolerance to saline–alkaline stress in rice (*Oryza sativa* L.). Crop J. 10, 354–367. doi: 10.1016/j.cj.2021.06.011

[ref76] Liu M. Yu H. Ouyang B. Shi C. Demidchik V. Hao Z.. (2020b). NADPH oxidases and the evolution of plant salinity tolerance. Plant Cell Environ. 43, 2957–2968. doi: 10.1111/pce.13907 33043459

[ref77] Lu C. Zhang J. Pan X.-B. Zhang F. Zheng T.-Q. Zhao X.-Q.. (2014). Advanced backcross QTL analysis for the whole plant growth duration salt tolerance in rice (*Oryza sativa* L.). J. Inte. Agricul. 13, 1609–1620. doi: 10.1016/S2095-3119(13)60575-4

[ref78] Lutts S. Kinet J. Bouharmont J. (1995). Changes in plant response to NaCl during development of rice (*Oryza sativa* L.) varieties differing in salinity resistance. J. Exp. Bot. 46, 1843–1852. doi: 10.1093/jxb/46.12.1843

[ref79] Maas E. (1996). “Crop salt tolerance,” in Agricultural Salinity Assessment and Management. ASCE Manuals and Reports on Engineering Practice (Virginia: American Society of Civil Engineers), 262–304.

[ref80] Mai N. S. Hanh D. D. Nakashima M. Kumamoto K. Thuy N. T. T. Kobata T.. (2021). Identification and validation of QTLs for yield and yield components under Long-term salt stress using IR64 CSSLs in the genetic background of Koshihikari and their backcross progenies. Agriculture 11:777. doi: 10.3390/agriculture11080777

[ref81] Manimaran P. Venkata Reddy S. Moin M. Raghurami Reddy M. Yugandhar P. Mohanraj S.. (2017). Activation-tagging in Indica rice identifies a novel transcription factor subunit, NF-YC13 associated with salt tolerance. Sci. Rep. 7, 1–16. doi: 10.1038/s41598-017-10022-9 28839256 PMC5570948

[ref82] Megha S. Shankhdhar D. (2022). Effect of different levels of salinity on morphological characteristics of some rice genotypes. Pha. Innov. J. 11, 189–193.

[ref83] Minh L. Khang D. Ha P. T. T. Tuyen P. T. Minh T. N. Quan N.. (2016). Effects of salinity stress on growth and phenolics of rice (*Oryza sativa* L.). Int. Lett. Nat. Sci. 57, 1–10. doi: 10.18052/www.scipress.com/ILNS.57.1

[ref84] Mishra S. Singh B. Panda K. Singh B. P. Singh N. Misra P.. (2016). Association of SNP haplotypes of HKT family genes with salt tolerance in Indian wild rice germplasm. Rice 9, 1–13. doi: 10.1186/s12284-016-0083-8 27025598 PMC4811800

[ref85] Mohammadinezhad G. Singh R. Arzani A. Rezaei A. Sabouri H. Gregorio G. (2010). Evaluation of salinity tolerance in rice genotypes. Int. J. Plant Produc. 4, 199–207.

[ref86] Moin M. Saha A. Bakshi A. Madhav M. Kirti P. (2021). Constitutive expression of ribosomal protein L6 modulates salt tolerance in rice transgenic plants. Gene 789:145670. doi: 10.1016/j.gene.2021.145670, PMID: 33892070

[ref87] Munns R. Tester M. (2008). Mechanisms of salinity tolerance. Annu. Rev. Plant Biol. 59, 651–681. doi: 10.1146/annurev.arplant.59.032607.092911, PMID: 18444910

[ref88] Nagarajan S. Varatharajan N. Gandhimeyyan R. V. (2022). “Understanding the responses, mechanism and development of salinity stress tolerant cultivars in Rice,” in Integrative Advances in Rice Research (IntechOpen).

[ref89] Nakhla W. R. Sun W. Fan K. Yang K. Zhang C. Yu S. (2021). Identification of QTLs for salt tolerance at the germination and seedling stages in rice. Plan. Theory 10:428. 10.3390/plants10030428 PMC7996262 33668277

[ref90] Nayyeripasand L. Garoosi G. A. Ahmadikhah A. (2021). Genome-wide association study (GWAS) to identify salt-tolerance QTLs carrying novel candidate genes in rice during early vegetative stage. Rice 14, 1–21. doi: 10.1186/s12284-020-00433-0 33420909 PMC7797017

[ref91] Nazir R. Mandal S. Mitra S. Ghorai M. Das N. Jha N. K.. (2022). Clustered regularly interspaced short palindromic repeats (CRISPR)/CRISPR-associated genome-editing toolkit to enhance salt stress tolerance in rice and wheat. Physiol. Plant. 174:e13642. doi: 10.1111/ppl.13642, PMID: 35099818

[ref92] Negrão S. Cecília Almadanim M. Pires I. S. Abreu I. A. Maroco J. Courtois B.. (2013). New allelic variants found in key rice salt-tolerance genes: an association study. Plant Biotech. J. 11, 87–100. doi: 10.1111/pbi.12010, PMID: 23116435

[ref93] Niu S. Gao Y. Zi H. Liu Y. Liu X. Xiong X.. (2022). The osmolyte-producing endophyte *Streptomyces albidoflavus* OsiLf-2 induces drought and salt tolerance in rice via a multi-level mechanism. Crop J. 10, 375–386. doi: 10.1016/j.cj.2021.06.008

[ref94] Nutan K. K. Singla-Pareek S. L. Pareek A. (2020). The Saltol QTL-localized transcription factor *OsGATA8* plays an important role in stress tolerance and seed development in Arabidopsis and rice. J. Exp. Bot. 71, 684–698. doi: 10.1093/jxb/erz368, PMID: 31613368

[ref95] Oomen R. J. Benito B. Sentenac H. Rodríguez-Navarro A. Talón M. Véry A. A.. (2012). HKT2; 2/1, a K+-permeable transporter identified in a salt-tolerant rice cultivar through surveys of natural genetic polymorphism. Plant J. 71, 750–762. doi: 10.1111/j.1365-313X.2012.05031.x, PMID: 22530609

[ref96] Pang Y. Chen K. Wang X. Wang W. Xu J. Ali J.. (2017). Simultaneous improvement and genetic dissection of salt tolerance of rice (*Oryza sativa* L.) by designed QTL pyramiding. Front. Plant Sci. 8:1275. doi: 10.3389/fpls.2017.01275 28775730 PMC5517400

[ref97] Pareek A. Dhankher O. P. Foyer C. H. (2020). Mitigating the impact of climate change on plant productivity and ecosystem sustainability. J. Exp. Bot. 71, 451–456. doi: 10.1093/jxb/erz518 31909813 PMC6945998

[ref98] Park H.-J. Seo B.-S. Jeong Y.-J. Yang H. I. Park S.-I. Baek N.. (2022). Soil salinity, fertility and carbon content, and rice yield of salt-affected paddy with different cultivation period in southwestern coastal area of South Korea. Soil Sci. Plant Nut. 68, 53–63. doi: 10.1080/00380768.2021.1967082

[ref99] Platten J. D. Egdane J. A. Ismail A. M. (2013). Salinity tolerance, Na+ exclusion and allele mining of HKT1; 5 in *Oryza sativa* and *O. glaberrima*: many sources, many genes, one mechanism? BMC Plant Biol. 13, 1–16. doi: 10.1186/1471-2229-13-32 23445750 PMC3599985

[ref100] Polash M. Sakil M. A. Tahjib-Ul-Arif M. Hossain M. A. (2018). Effect of salinity on osmolytes and relative water content of selected rice genotypes. Trop. Plant Res. 5, 227–232. doi: 10.22271/tpr.2018.v5.i2.029

[ref101] Prakash N. R. Lokeshkumar B. M. Rathor S. Warraich A. S. Yadav S. Vinaykumar N. M.. (2022). Meta-analysis and validation of genomic loci governing seedling and reproductive stage salinity tolerance in rice. Physiol. Plant. 174:e13629. doi: 10.1111/ppl.13629 35040153

[ref102] Pundir P. Devi A. Krishnamurthy S. Sharma P. C. Vinaykumar N. (2021). QTLs in salt rice variety CSR10 reveals salinity tolerance at reproductive stage. Acta Physiol. Plant. 43, 1–15. doi: 10.1007/s11738-020-03183-0

[ref103] Rahman M. A. Bimpong I. K. Bizimana J. Pascual E. D. Arceta M. Swamy B.. (2017). Mapping QTLs using a novel source of salinity tolerance from Hasawi and their interaction with environments in rice. Rice 10, 1–17. doi: 10.1186/s12284-017-0186-x 29098463 PMC5668218

[ref104] Rajendran K. Tester M. Roy S. J. (2009). Quantifying the three main components of salinity tolerance in cereals. Plant Cell Eenviron. 32, 237–249. doi: 10.1111/j.1365-3040.2008.01916.x 19054352

[ref105] Rasheed A. Fahad S. Aamer M. Hassan M. U. Tahir M. M. Wu Z. (2020b). Role of genetic factors in regulating cadmium uptake, transport and accumulation mechanisms and quantitative trait loci mapping in rice. A review. Appl. Ecol. Environ. Res. 18, 4005–4023. doi: 10.15666/aeer/1803_40054023

[ref106] Rasheed A. Fahad S. Hassan M. U. Tahir M. M. Aamer M. Wu Z. (2020a). A review on aluminum toxicity and quantitative trait loci maping in rice (*Oryza sativ*e L). App. Ecol. Environ. Res. 18, 3951–3961. doi: 10.15666/aeer/1803_39513964

[ref107] Rasheed A. Gill R. A. Hassan M. U. Mahmood A. Qari S. Zaman Q. U.. (2021a). A critical review: recent advancements in the use of CRISPR/Cas9 technology to enhance crops and alleviate global food crises. Curr. Issues Mol. Biol. 43, 1950–1976. 34889892 10.3390/cimb43030135 PMC8929161

[ref108] Rasheed A. Hassan M. U. Aamer M. Batool M. Sheng F. Ziming W.. (2020d). A critical review on the improvement of drought stress tolerance in rice (*Oryza sativa* L.). Notulae Bot. Horti Agrobot. Cluj-Nap. 48, 1756–1788. doi: 10.15835/nbha48412128

[ref109] Rasheed A. Hassan M. Aamer M. Bian J. Xu Z. He X.. (2020c). Iron toxicity, tolerance and quantitative trait loci mapping in rice; a review. App. Ecol. Environ. Res. 18, 7483–7498. doi: 10.15666/aeer/1806_74837498

[ref110] Rasheed A. Hassan M. U. Fahad S. Aamer M. Batool M. Ilyas M.. (2021b). “Heavy metals stress and plants defense responses,” in Sustainable Soil and Land Management and Climate Change (Abingdon, UK: Taylor and Francis Group), 57–82.

[ref111] Rasheed A. Wassan G. M. Khanzada H. Solangi A. M. Aamer M. Ruicai H.. (2021c). QTL underlying iron toxicity tolerance at seedling stage in backcross recombinant inbred lines (BRILs) population of rice using high density genetic map. Notulae Bot. Horti Agrobot. Cluj-Nap. 49:12158. doi: 10.15835/nbha49112158

[ref112] Rasheed A. Wassan G. M. Khanzada H. Solangi A. M. Han R. Li H.. (2021d). Identification of genomic regions at seedling related traits in response to aluminium toxicity using a new high-density genetic map in rice (*Oryza sativa* L.). Gen. Res. Crop Evol. 68, 1889–1903. doi: 10.1007/s10722-020-01103-2

[ref113] Reddy I. N. B. L. Kim B.-K. Yoon I.-S. Kim K.-H. Kwon T.-R. (2017). Salt tolerance in rice: focus on mechanisms and approaches. Rice Sci. 24:123–144. doi: 10.1016/j.rsci.2016.09.004

[ref114] Roy S. Mishra M. Kaur G. Singh S. Rawat N. Singh P.. (2022). *OsCyp2-P*, an auxin-responsive cyclophilin, regulates Ca^2+^ calmodulin interaction for an ion-mediated stress response in rice. Physiol. Plant. 174:e13631. doi: 10.1111/ppl.13631 35049071

[ref115] Roy S. J. Negrão S. Tester M. (2014). Salt resistant crop plants. Curr. Opinion Biotech. 26, 115–124. doi: 10.1016/j.copbio.2013.12.004, PMID: 24679267

[ref116] Sangwongchai W. Krusong K. Thitisaksakul M. (2022). Salt tolerance at vegetative stage is partially associated with changes in grain quality and starch physicochemical properties of rice exposed to salinity stress at reproductive stage. J. Sci. Food Agricul. 102, 370–382. doi: 10.1002/jsfa.11367 34139029

[ref117] Santosh Kumar V. Verma R. K. Yadav S. K. Yadav P. Watts A. Rao M.. (2020). CRISPR-Cas9 mediated genome editing of drought and salt tolerance (*OsDST*) gene in indica mega rice cultivar MTU1010. Physiol. Mol. Biol. Plants 26, 1099–1110. doi: 10.1007/s12298-020-00819-w, PMID: 32549675 PMC7266915

[ref118] Schmidt R. Schippers J. H. Welker A. Mieulet D. Guiderdoni E. Mueller-Roeber B. (2012). Transcription factor *OsHsfC1b* regulates salt tolerance and development in *Oryza sativa* ssp. japonica. AoB Plants. 2012:pls011. doi: 10.1093/aobpla/pls011 22616023 PMC3357053

[ref119] Shahid S. A. Zaman M. Heng L. (2018). “Soil salinity: historical perspectives and a world overview of the problem,” in Guideline for Salinity Assessment, Mitigation and Adaptation Using Nuclear and Related Techniques (Cham, Switzerland: Springer Nature Switzerland), 43–53.

[ref120] Singh R. K. Kota S. Flowers T. J. (2021). Salt tolerance in rice: seedling and reproductive stage QTL mapping come of age. Theoret. App. Gen. 134, 3495–3533. doi: 10.1007/s00122-021-03890-3, PMID: 34287681 PMC8519845

[ref121] Singh R. Mishra B. Singh K. (2004). Salt tolerant rice varieties and their role in reclamation programme in Uttar Pradesh. Ind. Far. 2, 6–10.

[ref122] Soares F. A. Chiangmai P. N. Laosutthipong C. (2021). Effect of salinity on agronomic characteristics of three rice varieties (*Oryza sativa* L.) at tillering stage. RSU Int. Res. Conference 584–592. doi: 10.14458/RSU.res.2021.142

[ref123] Solis C. A. Yong M.-T. Zhou M. Venkataraman G. Shabala L. Holford P.. (2022). Evolutionary significance of NHX family and NHX1 in salinity stress adaptation in the genus oryza. Int. J. Mol. Sci. 23:2092. doi: 10.3390/ijms23042092, PMID: 35216206 PMC8879705

[ref124] Tang Y. Bao X. Zhi Y. Wu Q. Guo Y. Yin X.. (2019). Overexpression of a MYB family gene, *OsMYB6*, increases drought and salinity stress tolerance in transgenic rice. Front. Plant Sci. 10:168. doi: 10.3389/fpls.2019.00168 30833955 PMC6387972

[ref125] Teng Y. Lv M. Zhang X. Cai M. Chen T. (2022). *BEAR1*, a bHLH transcription factor, controls salt response genes to regulate rice salt response. J. Plant Biol. 65, 217–230. doi: 10.1007/s12374-022-09347-4

[ref126] Tiwari S. Sl K. Kumar V. Singh B. Rao A. Mithra Sv A.. (2016). Mapping QTLs for salt tolerance in rice (*Oryza sativa* L.) by bulked segregant analysis of recombinant inbred lines using 50K SNP chip. PLoS One 11:e0153610. doi: 10.1371/journal.pone.0153610, PMID: 27077373 PMC4831760

[ref127] Todaka D. Nakashima K. Shinozaki K. Yamaguchi-Shinozaki K. (2012). Toward understanding transcriptional regulatory networks in abiotic stress responses and tolerance in rice. Rice 5, 1–9. doi: 10.1186/1939-8433-5-6 24764506 PMC3834508

[ref128] Tsai Y.-C. Chen K.-C. Cheng T.-S. Lee C. Lin S.-H. Tung C.-W. (2019). Chlorophyll fluorescence analysis in diverse rice varieties reveals the positive correlation between the seedlings salt tolerance and photosynthetic efficiency. BMC Plant Biol. 19, 1–17. doi: 10.1186/s12870-019-1983-8 31519149 PMC6743182

[ref129] Wang Z. Cheng J. Chen Z. Huang J. Bao Y. Wang J.. (2012b). Identification of QTLs with main, epistatic and QTL× environment interaction effects for salt tolerance in rice seedlings under different salinity conditions. Theoret. App. Gen. 125, 807–815. doi: 10.1007/s00425-021-03802-1 22678666

[ref130] Wang X. He Y. Wei H. Wang L. (2021). A clock regulatory module is required for salt tolerance and control of heading date in rice. Plant Cell Environ. 44, 3283–3301. doi: 10.1111/pce.14167, PMID: 34402093

[ref131] Wang R. Jing W. Xiao L. Jin Y. Shen L. Zhang W. (2015). The rice high-affinity potassium transporter1; 1 is involved in salt tolerance and regulated by an MYB-type transcription factor. Plant Physiol. 168, 1076–1090. doi: 10.1104/pp.15.00298, PMID: 25991736 PMC4741328

[ref132] Wang W.-C. Lin T.-C. Kieber J. Tsai Y.-C. (2019). Response regulators 9 and 10 negatively regulate salinity tolerance in rice. Plant Cell Physiol. 60, 2549–2563. doi: 10.1093/pcp/pcz149, PMID: 31359043

[ref133] Wang X. Ren P. Ji L. Zhu B. Xie G. (2022). *OsVDE*, a xanthophyll cycle key enzyme, mediates abscisic acid biosynthesis and negatively regulates salinity tolerance in rice. Planta 255, 1–15. doi: 10.1007/s00425-021-03802-1 34842977

[ref134] Wang H. Zhang M. Guo R. Shi D. Liu B. Lin X.. (2012a). Effects of salt stress on ion balance and nitrogen metabolism of old and young leaves in rice (*Oryza sativa* L.). BMC Plant Biol. 12, 1–11. doi: 10.1186/1471-2229-12-194 23082824 PMC3496643

[ref135] Wei H. Wang X. He Y. Xu H. Wang L. (2021). Clock component *OsPRR73* positively regulates rice salt tolerance by modulating *OsHKT2*; 1-mediated sodium homeostasis. EMBO J. 40:e105086. doi: 10.15252/embj.2020105086, PMID: 33347628 PMC7849171

[ref136] Wu J. Yu C. Huang L. Gan Y. (2021). A rice transcription factor, *OsMADS57*, positively regulates high salinity tolerance in transgenic Arabidopsis thaliana and *Oryza sativa* plants. Physiol. Plant. 173, 1120–1135. doi: 10.1111/ppl.13508, PMID: 34287928

[ref137] Wu J. Yu C. Hunag L. Wu M. Liu B. Liu Y.. (2020). Overexpression of MADS-box transcription factor *OsMADS25* enhances salt stress tolerance in Rice and Arabidopsis. Plant Growth Reg. 90, 163–171. doi: 10.1007/s10725-019-00539-6

[ref138] Xie L. Zheng C. Li W. Pu M. Zhou G. Sun W.. (2021). Mapping and identification a salt-tolerant QTL in a salt-resistant rice landrace, Haidao86. J. Plant Growth Reg. 1–12.

[ref139] Xu N. Chu Y. Chen H. Li X. Wu Q. Jin L.. (2018). Rice transcription factor *OsMADS25* modulates root growth and confers salinity tolerance via the ABA–mediated regulatory pathway and ROS scavenging. PLoS Genet. 14:e1007662. doi: 10.1371/journal.pgen.1007662, PMID: 30303953 PMC6197697

[ref140] Xu G. Cui Y. Wang M. Li M. Yin X. Xia X. (2014). *OsMsr9*, a novel putative rice F-box containing protein, confers enhanced salt tolerance in transgenic rice and Arabidopsis. Mol. Breed. 34, 1055–1064. doi: 10.1007/s11032-014-0096-1

[ref141] Xu C. Luo M. Sun X. Yan J. Shi H. Yan H.. (2022). *SiMYB19* from foxtail millet (*Setaria italica*) confers transgenic rice tolerance to high salt stress in the field. Int. J. Mol. Sci. 23:756. doi: 10.3390/ijms23020756, PMID: 35054940 PMC8775554

[ref142] Yadav A. K. Kumar A. Grover N. Ellur R. K. Bollinedi H. Krishnan S. G.. (2021). Genome-wide association study reveals marker–trait associations for early vegetative stage salinity tolerance in rice. Plants Theory 10:559. doi: 10.3390/plants10030559 PMC8000697 33809618

[ref143] Yan L. Baoxiang W. Jingfang L. Zhiguang S. Ming C. Yungao X.. (2021). A novel SAPK10-WRKY87-ABF1 biological pathway synergistically enhance abiotic stress tolerance in transgenic rice (*Oryza sativa*). Plant Physiol. Biochem. 168, 252–262. doi: 10.1016/j.plaphy.2021.10.006, PMID: 34656861

[ref144] Yang M. Lu K. Zhao F.-J. Xie W. Ramakrishna P. Wang G.. (2018). Genetic basis of rice ionomic variation revealed by genome-wide association studies. Plant Cell 30, 2720–2740. doi: 10.1105/tpc.18.00375, PMID: 30373760 PMC6305983

[ref145] Yarra R. Wei W. (2021). The NAC-type transcription factor *GmNAC20* improves cold, salinity tolerance, and lateral root formation in transgenic rice plants. Funct. Inte. Gen. 21, 473–487. doi: 10.1007/s10142-021-00790-z, PMID: 34191184

[ref146] Yeo A. Flowers T. (1990). Screening of rice genotypes for physiological character contributing to salinity resistance and their relationship to overall performance. Theor. Appl. Genet. 79, 277–384. doi: 10.1007/BF01186082 24226357

[ref147] Yin W. Xiao Y. Niu M. Meng W. Li L. Zhang X.. (2020). ARGONAUTE2 enhances grain length and salt tolerance by activating BIG GRAIN3 to modulate cytokinin distribution in rice. Plant Cell 32, 2292–2306. doi: 10.1105/tpc.19.00542, PMID: 32409321 PMC7346564

[ref148] Yuan L. Zhang L. Wei X. Wang R. Li N. Chen G.. (2022). Quantitative trait locus mapping of salt tolerance in wild rice *Oryza longistaminata*. Int. J. Mol. Sci. 23:2379. doi: 10.3390/ijms23042379, PMID: 35216499 PMC8878134

[ref149] Yue E. Cao H. Liu B. (2020). OsmiR535, a potential genetic editing target for drought and salinity stress tolerance in *Oryza sativa*. Plan. Theory 9:1337. doi: 10.3390/plants9101337 PMC7601473 33050518

[ref150] Zahra N. Al Hinai M. S. Hafeez M. B. Rehman A. Wahid A. Siddique K. H.. (2022). Regulation of photosynthesis under salt stress and associated tolerance mechanisms. Plant Physiol. Biochem. 178, 55–69. doi: 10.1016/j.plaphy.2022.03.003, PMID: 35276596

[ref151] Zegeye W. A. Tsegaw M. Zhang Y. Cao L. (2022). CRISPR-based genome editing: advancements and opportunities for Rice improvement. Int. J. Mol. Sci. 23:4454. doi: 10.3390/ijms23084454, PMID: 35457271 PMC9027422

[ref152] Zeng L. Shannon M. Grieve C. (2002). Evaluation of salt tolerance in rice genotypes by multiple agronomic parameters. Euphytica 127, 235–245. doi: 10.1023/A:1020262932277, PMID: 35684234

[ref153] Zeng D.-D. Yang C.-C. Qin R. Alamin M. Yue E.-K. Jin X.-L.. (2018). A guanine insert in *OsBBS1* leads to early leaf senescence and salt stress sensitivity in rice (*Oryza sativa* L.). Plant Cell Rep. 37, 933–946. doi: 10.1007/s00299-018-2280-y, PMID: 29572657

[ref154] Zhang Z. Liu H. Sun C. Ma Q. Bu H. Chong K.. (2018). A C2H2 zinc-finger protein *OsZFP213* interacts with *OsMAPK3* to enhance salt tolerance in rice. J. Plant Physiol. 229, 100–110. doi: 10.1016/j.jplph.2018.07.003, PMID: 30055519

[ref155] Zhang A. Liu Y. Wang F. Li T. Chen Z. Kong D.. (2019a). Enhanced rice salinity tolerance via CRISPR/Cas9-targeted mutagenesis of the *OsRR22* gene. Mol. Breed. 39, 1–10. doi: 10.1007/s11032-019-0954-y PMC7413041 32803201

[ref156] Zhang X. Long Y. Chen X. Zhang B. Xin Y. Li L.. (2021). A NAC transcription factor *OsNAC3* positively regulates ABA response and salt tolerance in rice. BMC Plant Biol. 21, 1–13. doi: 10.1186/s12870-021-03333-7 34800972 PMC8605558

[ref157] Zhang X. Long Y. Huang J. Xia J. (2020). *OsNAC45* is involved in ABA response and salt tolerance in rice. Rice 13, 1–13. doi: 10.1186/s12284-020-00440-1 33284415 PMC7721851

[ref158] Zhang C. Srivastava A. K. Sadanandom A. (2019b). Targeted mutagenesis of the SUMO protease, overly tolerant to Salt1 in rice through CRISPR/Cas9-mediated genome editing reveals a major role of this SUMO protease in salt tolerance. *BioRxiv* [Epub ahead of preprint].

[ref159] Zhang J. Sun Y. Zhou Z. Zhang Y. Yang Y. Zan X.. (2022). *OsSCL30* overexpression reduces the tolerance of rice seedlings to low temperature, drought and salt. Sci. Rep. 12, 1–15. doi: 10.1038/s41598-022-12438-4 35589923 PMC9120446

[ref160] Zhou Y. Xu S. Jiang N. Zhao X. Bai Z. Liu J.. (2022). Engineering of rice varieties with enhanced resistances to both blast and bacterial blight diseases via CRISPR/Cas9. Plant Biotech. J. 20, 876–885. doi: 10.1111/pbi.13766, PMID: 34890109 PMC9055821

[ref161] Zhou J. Yuan M. Zhao Y. Quan Q. Yu D. Yang H.. (2021). Efficient deletion of multiple circle RNA loci by CRISPR-Cas9 reveals Os06circ02797 as a putative sponge for *OsMIR408* in rice. Plant Biotech. J. 19, 1240–1252. doi: 10.1111/pbi.13544, PMID: 33440058 PMC8196656

